# Changes in the vaginal microbiota across a gradient of urbanization

**DOI:** 10.1038/s41598-020-69111-x

**Published:** 2020-07-27

**Authors:** Daniela Vargas-Robles, Natalia Morales, Iveth Rodríguez, Tahidid Nieves, Filipa Godoy-Vitorino, Luis David Alcaraz, María-Eglée Pérez, Jacques Ravel, Larry J. Forney, María Gloria Domínguez-Bello

**Affiliations:** 1 Department of Biology, University of Puerto Rico, San Juan, PR USA; 2 Servicio Autónomo Centro Amazónico de Investigación y Control de Enfermedades Tropicales Simón Bolívar, MPPS, Puerto Ayacucho, Venezuela; 3 Ministerio del Poder Popular Para La Salud, Caracas, Venezuela; 4 Department of Microbiology & Medical Zoology, School of Medicine, University of Puerto Rico, San Juan, PR USA; 5 Departamento de Biología Celular, Facultad de Ciencias, Universidad Nacional Autónoma de México, Mexico City, Mexico; 6 Department of Mathematics, University of Puerto Rico, San Juan, PR USA; 7 Institute for Genome Sciences, University of Maryland School of Medicine, Baltimore, USA; 8 Department of Biological Sciences and the Institute for Bioinformatics and Evolutionary Studies, University of Idaho, Moscow, ID USA; 9 Department of Medicine, New York University School of Medicine, New York, USA; 10 Departments of Biochemistry and Microbiology and of Anthropology, Rutgers University, New Brunswick, USA

**Keywords:** Microbial ecology, Population screening

## Abstract

The vaginal microbiota of healthy women typically has low diversity, which increases after perturbations. Among these, lifestyle associated with certain sexual and antimicrobial practices may be associated with higher diversity. To test this hypothesis, we characterized the vaginal microbiota in the cervicovaginal and introital sites in sexually active Amerindians (N = 82) spanning urbanization, and in urban mestizos (N = 29), in the Venezuelan Amazonas. HPV status was also considered. Sampling was performed in an urban gradient from remote villages to a town, and women were individually classified by the degree of urbanization (low, medium, and high). Amerindian cervicovaginal and introital microbiota diversity were not associated with major changes in urbanization or ethnicity. There was a non-significant trend of increased diversity with urbanization, with a few taxa found overrepresented in urban Amerindians (*Brevibacterium linens* and *Peptoniphilus lacrimalis*) or mestizos (*Mobiluncus mulieris* and *Prevotella sp.*). Among all women, cervicovaginal and introital samples clustered, respectively, in four and two community state types (CSTs), where most profiles were dominated by *Lactobacillus iners, Gardnerella vaginalis* or were highly diverse profiles. HPV status did not associate with microbial diversity. In conclusion, no association was found between urban level and the vaginal microbiome in Amerindian women, and little difference was found between ethnicities. *L. iners* and high diversity profiles, associated with vaginal health outcomes, prevail in these populations.

## Introduction

Typically, the vaginal microbiota of healthy women has low diversity in relation to other locations on the body^1^. This diversity is consistent with the low pH (< 4.5^2,3^) maintained by lactic acid produced by the different predominant *Lactobacillus* species, which is thought to restrict colonization of vaginal pathogens^4,5^. However, not all *Lactobacillus* are considered equally beneficial^6^.

Decrease in *Lactobacillus* spp. Abundance is often associated with a condition commonly known as bacterial vaginosis (BV)^2^. Bacterial vaginosis is associated with symptoms such as fishy odor discharge and discomfort, but some women show BV while remaining asymptomatic^7^. Importantly, vaginal profiles with high bacterial diversity has been associated with increased risk for pelvic inflammatory diseases^8^, preterm births^9,10^, and sexually transmitted infections (HIV^11^, herpes simplex virus type 2^12^ and human papillomavirus (HPV)^11,13–18^. Increased vaginal bacterial diversity has been reported after antibiotic therapy ^19^ and personal vaginal hygiene practices^20,21^, some sex practices^22,23^, high fat diet^24,25^, smoking^26^, and other factors that may vary across lifestyles. Despite the protection associated with high proportions of vaginal *Lactobacillus,* profiles dominated by *Lactobacillus iners* have been associated with increased risk of *Chlamydia trachomatis* infection^27^. Additionally, differences in the vaginal microbiota have also been associated with ethnicity (which is a composite of genetic and the behavioral attributes); Blacks and Hispanics have higher vaginal microbiota diversity^28^ than Caucasian and Asian women^29–32^.

Most studies on vaginal microbiota have focused on urban women in developed countries^15,29–33^, and only a few refer to women from urban^34^ or rural areas in developing countries^11,35^. Amerindians -peoples of Asian descent, from migrants that expanded in the Americas 14,000–24,000 years ago^36,37^, have remained in isolation (with genetic drift leading to genetic divergence^38–42^). The arrival of Europeans and Africans in the colonial times led to admixture^43^, resulting in the current mestizo population predominant in South America. Yet, there are still villages of isolated Amazonian communities living pre-agricultural lifestyles, with low frequency of medical visits, no running water or electric services, no market economy, and subsisting on fishing, hunting, and agriculture practices. Increased access to jobs, education and healthcare services have led to migration to urban areas thus starting a process of urbanization. In addition to the isolated and the urban Amerindians, other communities are in transition, with intermediate access to healthcare, public services and industrialized products. This transition occurs in a heterogeneous fashion within communities, with different individuals exposed differently. For example, in communities with low exposure to urban settings, a few villagers may be the ones who go to the urban locations, with incentives (teachers, nurses), or to sell their products in markets and slowly adopt urban practices.

Since different lifestyle practices and ethnicities may influence human vaginal microbiota, we decided to explore the vaginal microbiota of Amerindians in a gradient of urbanization, and between Amerindians with mestizos. In this study, we compared cervicovaginal and introital microbiota in Amerindian Piaroa women by urban status, and with mestizo women living in a town. Additionally, we considered HPV infection status in the analysis.

## Results

**Figure 1:**
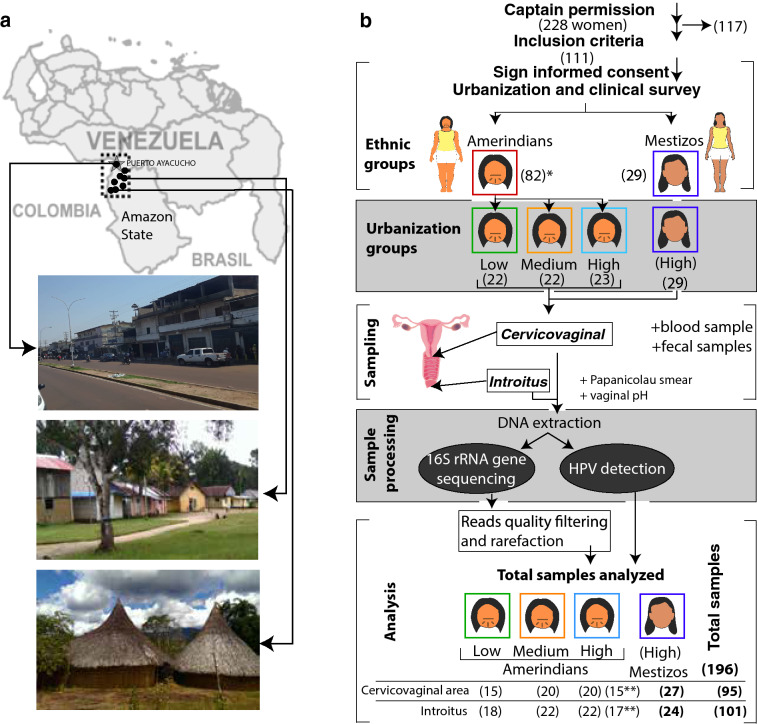
Study design. (**a**) Diagram of geographic locations (black points in the map) of the 7 villages and the urban town (Puerto Ayacucho, indicated with a star) of the study. Pictures depict the town (top) and Piaroa communities with intermediate and low access to urban services (middle and bottom pictures, respectively). (**b**) Numbers of women recruited, urban stratification, sampling, and sample analyses. In traditional communities, permission by the captain (Chief) preceded individual consent. In the urban town there was a public invitation to participate. From 228 women, who received a gynecological evaluation, 111 agreed to participate and complied with the inclusion criteria. A total of 82 Amerindians and 29 mestizos were included in the study. Surveys to assess urbanization status and clinical conditions were applied. A gynecologist sampled vaginal introitus and cervicovaginal (endo/ectocervix/posterior fornix) sites, using sterile swabs; Papanicolaou smear was performed and vaginal pH was taken. Additionally, blood for HIV, hemoglobin, hepatitis diagnostic, and fecal samples for parasite detection were also collected for ancillary studies. DNA was extracted from swabs, and used for human papillomavirus (HPV) detection and amplification of the regions V1-V3 of the 16S rRNA gene, which was then sequenced with Illumina MiSeq.

The study included 7 different communities of the Venezuelan Amazonas state (Fig. 1a). A total of 228 women received medical services. Out of this sample, 111 (49%) sexually active volunteers complied with the inclusion criteria (see methods; Fig. 1b). The mean age of the participants was 28.9 years (12 to 53 years old).

There were marked differences in lifestyle among Amerindians urbanization groups. These differences included an increment with urbanization of industrialized food consumption and education and sexual contact with mestizo men and a decrement with urbanization of crop gardening practices and number of pregnancies (Table S1). When looking at health variables, body mass index increased with urbanization (Table S2).

Crop gardening practices were more frequent in Amerindians from the high urbanization group than in mestizos. Mestizo women also reported having sexual practices other than vaginal, sexual contact with mestizo men, and use of vaginal douche (Table S1). All women reported to have monogamous practices, and do not practice female or male circumcision.

### Cervicovaginal and introital microbiota

Samples were taken by an MD at the cervicovaginal and introital vaginal sites, and immediately frozen until analyses. DNA was extracted and the region V1-V3 of the 16S rRNA was amplified and sequenced. A total 2,771,167 sequences (13,196 sequences/sample) were obtained from 222 cervicovaginal and introital samples from 111 women (eight environmental controls generated only 152 sequences). The low sequence number and the pattern in the environmental controls suggest that there was not a significant effect of contamination on the composition of the human samples. All environmental control samples were lost after rarefying at 1,655 sequences/sample. In addition, 25 (11%) samples with a low sequence number were also lost. The total number of samples analyzed was 196: 95 cervicovaginal and 101 introital (Fig. 1b, Table S3).

Amplicon Sequence Variants (ASVs) tables generated 1,015 different ASVs across all samples (mean of 15.4 and 20.1 ASVs for cervicovaginal and introital samples, respectively, Table S3). ASVs assigned to the same taxonomy were combined and this final table was used for the analysis. The average observed and estimated ASVs taxa per sample were respectively 9.19 and 9.32, showing a good sampling coverage of 92% according to Chao1. A total of 115 cervicovaginal and 161 introital ASV taxa were identified (Table S3), of which interestingly, 18 matched unidentified bacteria (Table S4), and 8 had sequence identity < 97% to sequences in non-redundant nucleotide NCBI database using the megaBLAST algorithm.

**Figure 2:**
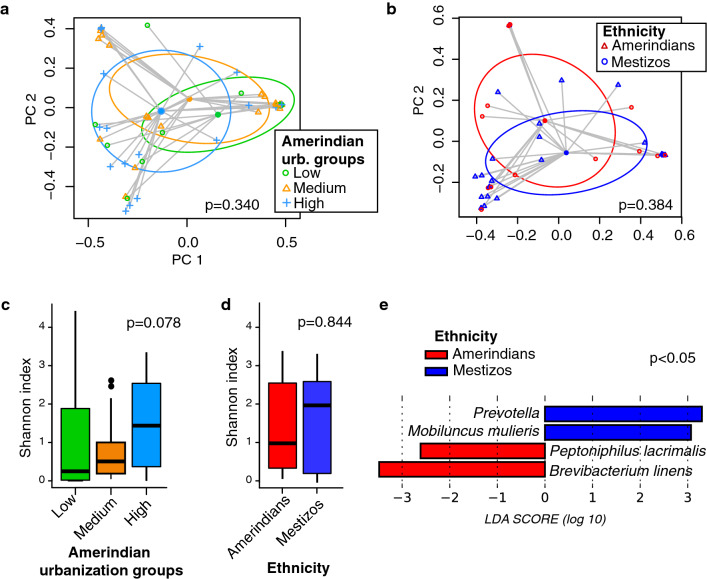
Cervicovaginal microbiota diversity. (**a**,**b)** Principal Coordinate Analysis (PCoA) of Bray–Curtis dissimilarity for Amerindians among urbanization groups (low n = 15, medium n = 20, high n = 20) (**a**), and between ethnicities: Amerindians n = 15, mestizo n = 27, (PERMANOVA) (**b**). Gray lines connect samples with the group centroid. Ellipses indicate one standard deviation. (**c**,**d**) Alpha diversity using Shannon index among Amerindian urbanization groups (linear mixed-effect model, LMM) (**c**), and between ethnicities (Kruskal–Wallis test) (**b**). (**e**) Discriminant taxa analysis between ethnicities (LEfSe).

Beta diversity comparisons among all women groups (Amerindian urbanization groups and mestizos) showed significant differences for both vaginal sites using unweighted UniFrac distances (p > 0.024, PERMANOVA, cervicovaginal: R^2^ = 0.10, and introital: R^2^ = 0.09; Fig. S1a) or marginally significant for Bray Curtis dissimilarities (p > 0.057, PERMANOVA, R^2^ = 0.062; Fig S1a). However, since ethnicity and location might be confounding factors, comparisons were conducted only among the Amerindian group controlling by village. Fewer differences were found across the urbanization gradient by any of the vaginal sites (p > 0.340, Bray Curtis dissimilarity, PERMANOVA, cervicovaginal: R^2^ = 0.056, and introital: R^2^ = 0.037; Fig. 2a,b, Table S5). Alpha diversity comparisons among all groups of women showed marginal differences which were lost after p value adjustment for multiple comparisons (p = 0.045, p.adj = 0.135, Faith PD; p > 0.108, for Shannon or Simpson; Fig. S1c). Analyses using a linear mixed-effect model randomizing the location variable and conducted among only Amerindian groups showed a tendency of increasing with urbanization, which was not significant (p > 0.061, for Shannon and Simpson; linear mixed-effect model, LMM; Fig. 2c. Table S5).

There were significant vaginal beta diversity differences of ethnicity between Amerindians as a group, and mestizos (p < 0.030, unweighted UniFrac distance PERMANOVA, cervicovaginal: R^2^ = 0.036; and introital: R^2^ = 0.019; Fig. S1b) and marginal differences for Bray Curtis dissimilarity (p > 0.068, PERMANOVA, R^2^ = 0.022). However, analysis only including Amerindian and mestizo women at the high urban level and living in the same location was not significant in any vaginal site (p > 0.322, PERMANOVA, Bray Curtis dissimilarity; Table S5, Fig. 2d). Marginal or no alpha diversity differences were detected between ethnicities in any vaginal site (p > 0.066 for Faith PD, Shannon or Simpson, Kruskal–Wallis; Fig. S1c) and even fewer differences when only urban Amerindians and mestizo from the same location were compared (p > 0.771, for Shannon and Simpson; Kruskal–Wallis; Fig. 2c. Table S5).

Discriminant analysis between groups of women showed that the majority of the differences observed were between mestizo and Amerindian groups, for both vaginal sites (Fig. S2). Mestizos were particularly higher in anaerobic taxa. Comparisons among only Amerindian urban groups did not show any differential taxa (LEfSe, alpha = 0.05; Table S6). However, in terms of prevalence *Dialister micraerophilus* increased with urbanization (0%, 5% and 30% for low, medium and high urbanization groups respectively; p = 0.021, Fisher´s exact test, Table S7). Discriminant taxa between ethnicities controlling for urbanization and location, showed in Amerindians significantly lower abundance of *Prevotella* and *Mobiluncus mulieris*, and higher *Peptoniphilus lacrimalis* and *Brevibacterium linens* compared to mestizo (LEfSe, alpha = 0.05; Fig. 2e); interestingly, *Mobiluncus mulieris*, was uniquely present in mestizo (in 7/27 women). Taxon prevalence also supported these results (Table S7). No discriminant taxon was found for introital samples between groups of women. A total of 30 taxa were shared among Amerindians from all urbanization levels, and 42 taxa were shared among urban Amerindians and mestizos; 19 taxa were uniquely found in Amerindians and 16 taxa in mestizos (Table S9). In general, most of the group’s unique taxa were found in only one or two women.

**Figure 3:**
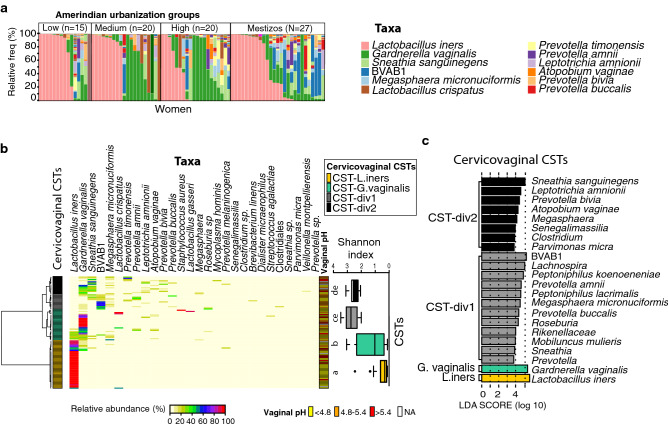
Cervicovaginal microbiota composition and community state type (CST) clustering. (**a**) Individual vaginal taxa plots; the legend shows the 12 most abundant taxa. (**b**) Heatmap of the 30 most abundant taxa by cervicovaginal CSTs resulting from the hierarchical clustering analysis (see text for explanation). Right hand boxplot shows Shannon index for each cervicovaginal CSTs: CST-*L. iners* < CST-*G.vaginalis* < CST-div2 = CST-div1. Different letters over the boxplots indicate significant differences (Kruskal–Wallis, p < 0.001). Vaginal pH is also shown. (**c**) Discriminant taxa for each cervicovaginal CSTs (LEfSe, p < 0.01).

Hierarchical clustering analysis of cervicovaginal samples yielded four clusters or community state types (CSTs; clustering Silhouette index value of 0.378, see methods; Fig. 3b,c): *L. iners* dominated (CST-*L. iners*), associated with lowest vaginal pH and alpha diversity (Table S9); *G. vaginalis* dominated (CST-*G. vaginalis*), associated with intermediate alpha diversity; and two highly diverse clusters, associated with highest vaginal pH and alpha diversity: CST-div1, enriched in bacterial vaginosis-associated bacterium-1 (BVAB1), *Lachnospira, Peptoniphilus koenoeneniae, Prevotella amnii*, among others; and CST-div2, enriched in *Sneathia sanguinegens, Leptotrichia amnionii, Prevotella bivia, Atopobium vaginae,* among others.

**Figure 4:**
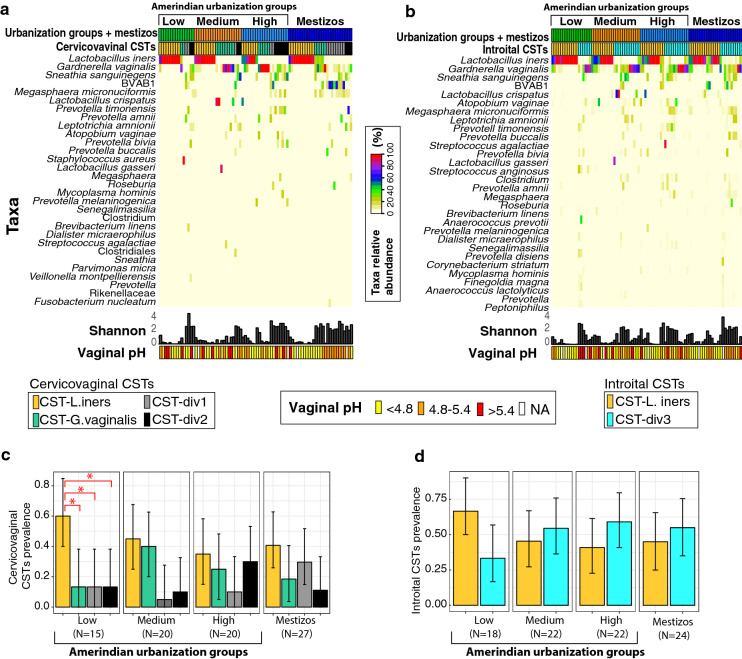
Bacterial community state types (CSTs) from cervicovaginal and introital sites in Amerindian urbanization groups and mestizos. (**a**,**b**) Heatmap of the 30 most abundant taxa in cervicovaginal (**a**) and introital (**b**) microbiota. There were no significant differences among groups for Shannon index and vaginal pH, shown at the bottom of the heatmaps. (**c**,**d**) Prevalence for each of the four cervicovaginal (**c**) and two introital (**d**) CSTs, by woman group. Confidence intervals of 95% are indicated in each bar. Although no association with urbanization was found for CST (log-linear model), comparison at the interior of each urbanization group showed significant differences (*) in the low urbanization groups (Fisher’s exact test); but not for the introital CSTs. NA indicates missing value.

Although the proportions of cervicovaginal CSTs were not associated with urbanization level or ethnicity (p > 0.318, log-linear model, ANOVA), the low-pH-associated cervicovaginal CST-*L. iners* was more prevalent than other CSTs in the low urbanization group (p = 0.001, Fisher’s exact test; p = 0.042, post hoc test; Fig. 4a,c). On the other hand, introital samples clustered in only two groups (clustering Silhouette index value of 0.466, see methods): (1) the low pH- low diversity *L. iners* dominated (CST-*L. iners*); and CST-div3, a high diversity cluster enriched in *Gardnerella vaginalis, Sneathia sanguinegens, Porphyromonas uenonis* among others (Fig. S3 a,b). There were no significant differences in introital CSTs with urbanization or ethnicity (p > 0.317, log-linear model, ANOVA; Fig. 4b,d).

Lifestyle, health, and demographic variables (Tables S1 and S2) were analyzed for CSTs association (Fisher’s exact test, Table S9). Crop gardening, a surrogate of traditional lifestyle, was associated with low-diversity CSTs (cervicovaginal CST-*L. iners* and CST-*G. vaginalis;* p < 0.001, log-linear model). Cytological smears with BV signs (≥ 20% of clue cells) was associated with high diversity cervicovaginal CST-div1 (p < 0.001, log-linear model).

Comparison between the diversity of cervicovaginal and introital samples showed non- significant differences for beta (p = 0.243, R^2^ = 0.011, PERMANOVA; Fig. S3c), or alpha diversities (Shannon and Simpson index, p > 0.230, Kruskal–Wallis test; Fig. S3f). However, there was 78% congruency of bacterial community state types (such as CST-*L.iners* or not) in the cervicovaginal and introital sites of individual woman (moderate strength of agreement with a Cohen’s Kappa = 0.553; Fig. S3c). In Amerindians, *Dialister micraerophilus* and *Ureaplasma parvum* were significantly higher in the cervicovaginal than the introital microbiota, while in mestizo women *Anaerococcus christensenii* was higher in the introital than in the cervicovaginal site (p < 0.050, LEfSe; Fig. S3d). No discriminant taxa were observed between both vaginal sites within each urbanization group.

*Lactobacillus iners* followed by *Gardnerella vaginalis* were the most abundant taxa in the vaginal microbiota of these populations (Fig. 3a). Close to half of all women (46%) harbored cervicovaginal or introital microbiota dominated by *Lactobacillus* spp., with *L. iners* being the dominant species within the genus (45%, Fig. 3a, Table S11). However, as high as 70% of the cervicovaginal samples showed at least one *Lactobacillus* sp., although some with very low relative abundance (Table S11).

An independent analysis was performed among all ASVs classified as *L. iners*. From a total 90 *L. iners* ASVs, 43 were present in more than one woman. The three most prevalent *L. iners* ASVs were found in 66%, 5%, and 5% of women and the rest in < 2%. No association with urbanization, ethnicity and HPV infection were found for these three *L. iners* ASVs.

### HPV infection

Microbiota diversity did not differ significantly between positive *vs.* negative HPV samples, high-risk *vs.* no-high-risk HPV types or among only-high, only low *and* both risk HPV types (for beta diversity p > 0.495, PERMANOVA; Fig. S4a; for alpha diversity p > 0.380, Kruskal–Wallis test; Fig. S4d, Table S12). Additionally, no alpha diversity associations were detected for any of the HPV types (p > 0.050, Fig. S4e). However, discriminant taxa analysis showed a significantly higher relative abundance of Coriobacteriaceae and *Anaerococcus tetradius* associated with lack of HPV infection (p < 0.05, LEfSe; Fig. S4b). The relative abundance of *Prevotella amnii* was significantly higher in women infected only with high-risk HPV types (Fig. S4c), while *Prevotella,* was positively associated with HPV16 presence (data not shown; *ASVs classified up to genus Prevotella are other than those classified as Prevotella amnii*). Finally, HPV infection or HPV types were not associated with CSTs, not even considering two groups of microbiota profiles: *Lactobacillus-*dominated or non-*Lactobacillus* dominated, for both vaginal (p > 0.050, Fisher’s exact test).

## Discussion

Previous work has been done in the oral mucosa^44,45^, skin^45,46^, and gut^45,47^ microbiota of Amerindians and other populations, but this work is a pioneer in studying the vaginal microbiota in relation with lifestyle. With the current sample size and statistical power, this study shows little cervicovaginal microbial differences spanning urbanization (marginal to the non-significant tendency of increasing diversity with urbanization), with some taxa discriminating urban Amerindian from mestizos. The differences are consistent with differences in habits that might influence the vaginal microbiome (sex practices^22,23^, vaginal douching^20,21^, diet^24,25^, antibiotic use^19^, smoking^26^, etc.).

Important limitations of this work are the low sample size -due to difficult access to remote communities and small village sizes- and lack of genetic information to determine ethnicity -due to restrictions in the IRB permits-. Ethnicity may be an influential compositional factor, but genetic background and lifestyle are difficult to separate. There is an admixture level between Amerindians and mestizos. Based on an autosomal DNA genetic study, mestizos have a contribution of 67% European, 23% Amerindian, and 16% African^48^. Urban Amerindians included in this study may have none or lower admixture, since they have a short history of cohabitation with mestizos (~ 40 years), and inter-marriages are not common.

*L. iners-*dominated profiles, was highly prevalent in both vaginal sites in the women of this study. This profile is also prevalent in vaginal microbiota of in North American Hispanic, Asian and African-American women^29^. It is also found in women in Africa^35,49,50^ and in Hispanics from the Caribbean^51^. Other *Lactobacillus*-dominated vaginal profiles prevalent in other populations (such as > 15% *L. crispatus*, > 5% *L. gasseri*^29–31^), were rare in this study and did not form an independent cluster out of the hierarchical clustering. *L. crispatus-*dominant profile was reported common in Tokyo^31^. Cervicovaginal *L. crispatus* was not absent. Although it did not form an independent cluster, it was identified in 15% of the samples, and in 3.2% as the dominant taxon (> 50% of relative abundance). However, the differences between the CSTs observed in these populations merit further studies—using marker genes such as chaperonin-60 universal target, and comparing with other populations—to confirm that the healthy women harbor very low *L. crispatus.* No significant primer bias is expected as we amplified the V1-V3 hyper variable region of the 16S rRNA gene with primers including seven different 27F primer sequences to capture a broad spectrum of taxa, including *L. crispatus*^52–56^. We also found a relatively high prevalence of the low diversity *G. vaginalis*-dominated profile, previously reported as common among women^32,57^. The high prevalence of these two low diversity clusters is consistent with the low number of taxa described among this population (average of 9 taxa/woman). Additionally, the data showed two polybacterial clusters (high diversity CST) in the cervicovaginal site also described in other reports^29,58^. Small differences between vaginal sites were observed, with moderate congruence of CST-*L.iners* in both sites, and some discriminant taxa. Comparison between cervicovaginal and introital microbiota in previous studies showed similarities in some^59^ and differences in others^53,60^.

Certain bacterial profiles have implications on the temporal stability of the vaginal microbiota^58^. Ecologically, shifts from *L. iners*-dominated to non-*Lactobacillus* dominated profiles are more frequent than shifts to any other *Lactobacillus*-dominated profile^57,61,62^. Genomic and metabolic characteristics suggest that *L. iners* is better adapted to environmental changes, such as hormonal levels (reviewed in^6^). *L. iners* dominance might constitute an advantage for maintaining a *Lactobacillus*-dominated profile in women with frequent hormonal changes due to high pregnancy rates (mean of 4.4 pregnancy/woman in this study). If genetic factors are relevant, we could expect similarities between Amerindians with Asians, given the Asian ancestry, and indeed, other studies have shown high prevalence of *L. iners*-dominated vaginal profiles in Asians living in USA^29^ and South-Asian Surinamese^32^ women.

The low urbanization group had the highest prevalence of cervicovaginal CST-*L. iners* in relation to other CSTs. Interestingly, CST-*L. iners* and CST-*G. vaginalis* were positively associated with a surrogated marker of a traditional lifestyle, such as crop gardening, involving factors that may affect the microbiota, such as physical activity (reviewed in^63^) and frequent contact with rich-bacterial environments (reviewed in^64^).

Ethnicity comparisons were performed between high urban level Amerindians and mestizo women living in the same location. Ethnic groups may reflect both genetics and lifestyles; for example, even living in urban conditions, traditional practices may be culturally preserved (urban Amerindian women still maintain traditional crops, tend to have only vaginal intercourse with Amerindian men, and do not adopt practices such as vaginal douches, used by 42% of mestizo women). There were few discriminant species between Amerindian and mestizo women. Amerindians had high prevalence of *Brevibacterium linens*, an aerobic bacterium rarely reported in the human vagina, rather common in cheese (reviewed in^65^), and associated with preterm birth^66^. Amerindians also had high *Peptoniphilus lacrimalis*, related to persistent BV^67^. Mestizo had an overrepresentation of vaginal *Mobiluncus mulieris* and *Prevotella*, both typically associated with high diversity profiles^29^, linked to BV^68–71^. Both taxa have been previously correlated with high genital pro-inflammatory cytokine levels (IL-1α, IL-1β, TNF-α) reflecting an elicited immune response^62^. *Mobiluncus mulieris* seems to facilitate *G. vaginalis* and other BV-associated taxa growth (reviewed in^72^).

The high prevalence of HPV (77%) found in this population^73^ may be associated with the general lack of *L. crispatus*^6,14^. High HPV prevalence has been associated with high *L. iners* and *G. vaginalis*^17^ or with non-*Lactobacillus* profiles^18,74^, as opposed to *L. crispatus*^6,14^. High-risk HPV types are known to elicit inflammatory responses (IL-1α, IL-1β, and IL-8) from cervical epithelial cells^62^. In this study, HPV-16 was associated with *Prevotella amnii*, an association also reported previously^13^. The relationship between HPV and the vaginal microbiota might be important since high diversity has been associated with HPV infection and with cervical inflammation^18,74^, mucin-degrading activity (sialidases) and cytolysin (vaginolysin) that damage the epithelium^75,76^.

In summary, Amerindians and mestizo show vaginal profiles dominated by *L. iners*, by *G. vaginalis*, and high diversity profiles, with consistency between vaginal sites. A few taxa discriminated between urban Amerindian and mestizos and a trend to increase diversity with urbanization in Amerindians. Additional inquiries into host genetic information could improve our understanding of the factors underlying these changes and their significance for women’s health.

## Materials and methods

### Study design

This study is part of a larger project aimed at determining the effect of urbanization on the microbiota of Amerindians, of which we have published the prevalence and types vaginal HPVs^73^. Amazonian women including Amerindians and mestizo of the northern area of the Amazonas State, Venezuela, were invited to participate in the study. Protocols and informed consent were approved by SA *Centro Amazónico de Investigación y Control de Enfermedades Tropicales Simón Bolívar*, Venezuela (SACAICET, IRB #78-2014), and University of Puerto Rico (IRB #1314-163).

Amerindian women were from the ethnic group Piaroa (women with both parents Piaroa who were self-identified as such and speak Piaroa language). After the permission by the village Captain, each woman gave her informed consent for study participation (signing or stamping their fingerprint), for participants under 18 years old informed consent from a parent and/or legal guardian was obtained. Sexually active women, ranging from 12 to 53 years old and compliant with inclusion criteria were enrolled. Inclusion criteria included women of reproductive age who at the time of recruitment had none of the following: pregnancy, menses, bleeding in the last 24 h, sexually transmitted infection diagnosed in the last 2 months (for those who had medical access), antibiotics in the last month, vaginal douches in the last 24 h, sexual intercourse in the last 24 h, hysterectomy, diabetes, urinary incontinence, urinary tract infections, and HIV. Women were excluded (n = 117) mostly due to recent exposure to antibiotics or antiparasitic drugs (28%), menses (25%), post-menopausal (13%), pregnant (12%), urinary infections (8%), refusing to participate (4%), sexual contact in the last 24 h (3%), hysterectomy (2%), belonging to a different ethnicity (1%), diabetes (1%), and HIV (1%). One HIV positive patient was sampled, although she was removed from the study after HIV diagnosiss (she was already in treatment although she denied she was infected during the inclusion criteria questions). Those women who did not meet the inclusion criteria also received medical service. No information about the filial relationship among volunteers was recorded. However, a relative level of endogamy is expected, and it may to be particularly high in smaller Amerindian communities.

Eight communities (Autana municipality in Amazonas state) and one urban town (Puerto Ayacucho, Amazonas state’s capital) formed a gradient of urbanization and were chosen for the study (described previously^73^). Some communities were located deep in the rainforest, a 2 day walk from the closest rural medical post, hence with scarce contact with urban population. These remote communities have a medical visit once every 1–2 years for children compulsory vaccination (HPV vaccine has not been included in the public health program in Venezuela up to date), have no running water or electrical services, and subsist on fishing, hunting and agriculture practices. Other communities show intermediate exposure and others that would usually live in an urban town with health and other public services available and access to industrialized products (Fig. 1).

Clinical information (STI history), lifestyle habits (number of sexual partners, diet) including typical urban practices (use of vaginal douches, contraception, and antibiotic use) were recorded from the recruited volunteers. Due to the social taboo around sexual practices, no information about simultaneous coupling or same-sex couples was collected, however, the Piaroa culture defines itself as being monogamous. Women were classified in three levels, namely low, medium or high urbanization level based on their individual level of adoption or urban lifestyles of abandon of traditional habits as described previously (Urbanization survey^73^; Fig. 1). Individual-level of urbanization was selected rather than community-based urban level for a higher classification resolution^73^.

### Samples and sample processing

An obstetrics-gynecologist associated with the study team sampled two vaginal sites, the introitus and the endo/ectocervix and posterior fornix (referred here as cervicovaginal area), which was sampled last. Samples were obtained by rotating the area with a sterile cotton swab. Cervicovaginal samples were taken with the use of a disposable speculum with sterile saline solution for lubrication. Since samples were collected under various conditions, eight environmental controls were taken for each location visited exposing a swab for 20 s to the air. Swabs were stored up to 4 h on ice in sterile and empty tubes, followed by storage in liquid nitrogen. Papanicolaou smears were also taken. Smear results described for endo/ectocervical abnormalities followed the Bethesda 2001 classification system, and reported the presence of clue cells, consistent with diagnostic of BV^77^. Vaginal pH was measured by pressing an introital swab on a piece of pH paper and reading the color scale (range: 4–10, ColorPhast).

A drop of blood was taken from finger prick for in situ hemoglobin (using *Easylife* rapid test in peripheral blood)^78^, and serum tests were performed for HIV, syphilis, and hepatitis B and C at the Public Health Center of Puerto Ayacucho. No specific assay for *C. trachomatis* was performed. HIV and hepatitis positive patients were excluded from the study. Fecal samples collected by each woman were preserved in tubes with iodine-formaldehyde for microscopy analysis of intestinal parasites.

DNA was extracted from swabs and feces using MoBio (CA, USA) PowerSoil according to the instructions provided by the manufacturer. Extracted DNA from samples was stored at − 20 °C until sequencing. The V1-V3 region of 16S rRNA gene was amplified by PCR. Controls for reagent and DNA extractions were also amplified and sequenced. Amplification were ran in two rounds of PCR with dual barcode indexing using the primers 27F and 534R as described elsewhere^54^ although here a HotStar HiFidelity Polymerase (Qiagen) was used. Sequencing was performed on an Illumina MiSeq Instrument at the University of Idaho in 2017. Additionally, extracted DNA was used for determining cervicovaginal HPV status which has been previously studied for this same population^73^. The method used was reverse hybridization method SPF_10_-PCR- LiPA25 system, version 1 (Labo Biomedical Products, Rijswijk, The Netherlands, based on licensed Innogenetics technology), which allows to detect 25 of the most common mucosa HPV types (types 6, 11, 16, 18, 31, 33, 34, 35, 39, 40, 42, 43, 44, 45, 51, 52, 53, 54, 56, 58, 59, 66, 68/73, 70, and 74).

### Bioinformatic analyses

Paired-end sequences were merged using FLASH^79^ yielding a 460 bp fragment. Sequences were filtered for quality and Amplicon Sequence Variants (ASVs) were identified using DADA2^80^. ASVs classification to the species level was performed using SPecies level Identification of metaGenOmic amplicons (SPINGO^81^), with default settings. SPINGO is a software dedicated to high-resolution assignment of sequences to species level using partial 16S rRNA gene sequences. It uses a reference database built with full-length (≥ 1,200 bp) bacterial and archaeal 16S rRNA gene sequences obtained from the Ribosomal Database Project version 11.2^82^ and sequences are labeled to species names according to the NCBI taxonomy (https://www.ncbi.nlm.nih.gov/guide/taxonomy/). In this study, SPINGO classifications were accepted or rejected based on their similarity scores above 0.5 for species-level classifications. In cases where the similarity scores were below 0.5 for the SPINGO species and genus assignments, the *RDP naive Bayesian *classifier method and the Silva reference database were used to assign the ASV to a family or higher taxonomic rank. Eighteen ASVs were not classified and were searched against NCBI database nucleotide collection (nt) using megaBLAST^83^ and were labeled as their closest hit, ≥ 97% identity (*e.g.* Uncultured_bacterium_5669ncd431b01c1). Sequence counts per ASV that were assigned to the same taxa were combined to obtain the final abundance table. Sequences assigned to *Shuttleworthia* sp., were aligned manually against the NCBI database nucleotide collection (nr/nt) using megaBLAST and reassigned to BVBA-1, as previously described^84^.

ASVs abundance table was rarified at 1655 reads per samples. Bacterial sequences were analyzed using the QIIME 1.9.1 platform^85^ and R v.3.3.2 packages^86^. Urbanization group comparison was performed only among Amerindian women (low, n = 15–19; medium, n = 20–22; and high, n = 20–22 urbanization groups, N are for cervicovaginal and introital samples respectively), controlling for the location variable (women village). To make possible controlling for location, nearby communities (< 10 km of separation) were combined, for a total of 4 location areas. For ethnicities comparison urbanization and village variables were fixed, only including women from the high urbanization group and living in nearby villages (Puerto Ayacucho and Alto Carinagua, at 10 km or 20 min. from each other in public transportation; Amerindians, N = 15–17 and mestizo, N = 27–24; for cervicovaginal and introital samples respectively).

Beta diversity analysis was performed with Bray–Curtis dissimilarities. Adonis2 function from the *vegan*^87^ R package, was used to execute non-parametric Permutational Multivariate Analysis of Variance (PERMANOVA^88^) to compared variance between groups to the variance within groups (spatial location differences). Principal Coordinates Analysis (PCoA) plots were generated with *vegdist*, *betadisper,* and plot functions in R drawing one standard deviation ellipses by group. Alpha diversity was performed with Shannon and Simpson index fitting linear mixed-effect models for urbanization analyses (LMM) or with Kruskal–Wallis test for ethnicity comparisons. Linear Mixed-Effect Model (LMM, controlling for woman location) was performed using *lme4* package^89^ with log10 transformed data to reach normality in residues.

Bacterial cervicovaginal and introital community state types (CSTs) were generated by hierarchical clustering as described previously for vaginal samples^58^. Briefly, squared root of Jensen-Shannon divergence measure matrices from each woman including proportions of ASVs were calculated using the *textmineR* R package^90^. Hierarchical clustering was performed with *hclust* function and *ward.D* method. The number of clusters were defined using *clValid* package with local editions to allow the Jensen-Shannon dissimilarity (JSD) metric. The *clvalid* function was set to explore between 2 and 10 possible clusters with hierarchical clustering method, Ward´s agglomeration method and internal validation with Silhouette index (SI) ranging from − 1 (lowest confidence) to 1 (highest confidence)^91^. Additionally, prediction strength (PS)^92^ was measured with *Philentropy* R package^93^ using the same JSD matrix and hierarchical clustering. Cervicovaginal site yielded four clusters with a maximum SI of 0.378 and with a PS of 60% and for introital two clusters with a SI of 0.466 samples with the PS of 77%. However, two clusters were suggested SP when applying a criterion of ≥ 80% for cervicovaginal and no cluster for introital samples. SI validation method was chosen for the final number of clustering.

Sampling coverage was estimated using *iNext*^94^ R package, for datatype = "abundance", nboot = 999. A Venn diagram was build using an online tool (https://bioinformatics.psb.ugent.be/webtools/Venn/). Cohen’s kappa coefficient^95^ and percentage of coincidence among body site CSTs were calculated with GraphPad QuickCalcs Web (https://www.graphpad.com/quickcalcs/kappa2/) and was interpreted as: < 0 less than chance; 0.01–0.20 slight; 0.21– 0.40 fair; 0.41–0.60 moderate; 0.61–0.80 substantial; and 0.81–0.99 almost perfect agreement^96^. Discriminant taxa analyses were performed with LEfSe^97^. LEfSe analysis was performed, only for CSTs comparison, with one-against-all strategy.

To explore *L. iners* variants (16S rRNA gene amplicons sequences variants, ASVs, classified within *L. iners*) and possible associations with the study’s variables, three of the most prevalent *L. iners* ASVs were compared with urbanization, ethnicity and HPV infection using fisher’s exact test.

Statistical analyses for CSTs within urbanization groups included log-linear model with R base functions, Fisher’s exact test and pairwise Fisher's exact test, performed with *fmsb* R package^98^ for categorical variables. Lifestyle variable analyses were performed with ANOVA, TukeyHSD, Kruskal–Wallis and pairwise Kruskal–Wallis tests for continuous variables**.** Adjustment for multiple comparisons was performed using the Benjamini-Hochberg’s false discovery rate (FDR-BH) method^99^. Statistics and graphics were also performed using the *reshape2*^100^, *ggplot2*^100^, and *gplots*^101^ R packages. For body site comparisons, QIIME was used to build taxa bar plots and to visualize UniFrac distance matrices in PCoA plots through EMPeror^102^. QIIME was also used for taxa bar visualization.

## Supplementary information

Supplementary figures.

Supplementary tables.

## Data Availability

Sequences and their associated metadata that support this study have been deposit in NCBI with the Sequence Read Archive (SRA) submission number SUB4588816 and BioProject ID PRJNA505490.

## References

[1] Huttenhower C (2012). Structure, function and diversity of the healthy human microbiome. Nature.

[2] Nugent RP, Krohn MA, Hillier SL (1991). Reliability of diagnosing bacterial vaginosis is improved by a standardized method of gram stain interpretation. J Clin Microbiol.

[3] Amsel R (1983). Nonspecific vaginitis: diagnostic criteria and microbial and epidemiologic associations. Am. J. Med..

[4] Boris S, Barbes C (2000). Role played by lactobacilli in controlling the population of vaginal pathogens. Microbes Infect.

[5] Tyssen, D. *et al.* Anti-HIV-1 Activity of Lactic Acid in Human Cervicovaginal Fluid. *mSphere* **3**, e00055–00018 (2018). 10.1128/mSphere.00055-18 PMC6034077 29976641

[6] Petrova MI, Reid G, Vaneechoutte M, Lebeer S (2017). Lactobacillus iners: friend or foe?. Trends Microbiol..

[7] Schwebke JR, Desmond R (2007). Natural history of asymptomatic bacterial vaginosis in a high-risk group of women. Sex. Transm. Dis..

[8] Larsson P-G, Platz-Christensen J-J, Thejls H, Forsum U, Påhlson C (1992). Incidence of pelvic inflammatory disease after first-trimester legal abortion in women with bacterial vaginosis after treatment with metronidazole: a double-blind, randomized study. Am. J. Obstet. Gynecol..

[9] Hillier SL (1995). Association between bacterial vaginosis and preterm delivery of a low-birth-weight infant. N. Engl. J. Med..

[10] Donders G (2009). Predictive value for preterm birth of abnormal vaginal flora, bacterial vaginosis and aerobic vaginitis during the first trimester of pregnancy. BJOG.

[11] Borgdorff H (2014). Lactobacillus-dominated cervicovaginal microbiota associated with reduced HIV/STI prevalence and genital HIV viral load in African women. ISME J..

[12] Cherpes TL, Hillier SL, Meyn LA, Busch JL, Krohn MA (2008). A delicate balance: risk factors for acquisition of bacterial vaginosis include sexual activity, absence of hydrogen peroxide-producing lactobacilli, black race, and positive herpes simplex virus type 2 serology. Sex. Transm. Dis..

[13] Dareng EO (2016). Prevalent high-risk HPV infection and vaginal microbiota in Nigerian women. Epidemiol. Infect..

[14] Brotman RM (2014). Interplay between the temporal dynamics of the vaginal microbiota and human papillomavirus detection. J. Infect. Dis..

[15] Lee JE (2013). Association of the vaginal microbiota with human papillomavirus infection in a Korean twin cohort. PLoS ONE.

[16] Rodriguez-Cerdeira C, Sanchez-Blanco E, Alba A (2012). Evaluation of association between vaginal infections and high-risk human papillomavirus types in female sex workers in Spain. ISRN Obstet. Gynecol..

[17] Di Paola M (2017). Characterization of cervico-vaginal microbiota in women developing persistent high-risk Human Papillomavirus infection. Sci. Rep..

[18] Cauci S (2004). Vaginal immunity in bacterial vaginosis. Curr. Infect. Dis. Rep..

[19] Stokholm J (2014). Antibiotic use during pregnancy alters the commensal vaginal microbiota. Clin. Microbiol. Infect..

[20] Welch JS (1993). Quantitative and qualitative effects of douche preparations on vaginal microflora. Obstet. Gynecol..

[21] Brotman RM (2008). The effect of vaginal douching cessation on bacterial vaginosis: a pilot study. Am. J. Obstet. Gynecol..

[22] Schwebke JR, Richey CM, Weiss HL (1999). Correlation of behaviors with microbiological changes in vaginal flora. J. Infect. Dis..

[23] Beigi RH, Wiesenfeld HC, Hillier SL, Straw T, Krohn MA (2005). Factors associated with absence of H2O2-producing Lactobacillus among women with bacterial vaginosis. J. Infect. Dis..

[24] Ahluwalia N, Grandjean H (2007). Nutrition, an under-recognized factor in bacterial vaginosis. J. Nutr..

[25] Neggers YH (2007). Dietary intake of selected nutrients affects bacterial vaginosis in women. J. Nutr..

[26] Brotman RM (2014). Association between cigarette smoking and the vaginal microbiota: a pilot study. BMC Infect. Dis..

[27] van Houdt R (2017). *Lactobacillus iners*-dominated vaginal microbiota is associated with increased susceptibility to *Chlamydia trachomatis* infection in Dutch women: a case–control study. Sex. Transm. Infect..

[28] Noyes N, Cho K-C, Ravel J, Forney LJ, Abdo Z (2018). Associations between sexual habits, menstrual hygiene practices, demographics and the vaginal microbiome as revealed by Bayesian network analysis. PLoS ONE.

[29] Ravel J (2011). Vaginal microbiome of reproductive-age women. Proc. Natl. Acad. Sci. USA.

[30] Zhou X (2007). Differences in the composition of vaginal microbial communities found in healthy Caucasian and black women. ISME J..

[31] Zhou X (2010). The vaginal bacterial communities of Japanese women resemble those of women in other racial groups. FEMS Immunol. Med. Microbiol..

[32] Borgdorff H (2017). The association between ethnicity and vaginal microbiota composition in Amsterdam, the Netherlands. PLoS ONE.

[33] Verstraelen H (2016). Characterisation of the human uterine microbiome in non-pregnant women through deep sequencing of the V1–2 region of the 16S rRNA gene. PeerJ.

[34] Rick, A.-M. *et al.**Open forum infectious diseases.* (Oxford University Press, Oxford).

[35] Anukam KC, Osazuwa EO, Ahonkhai I, Reid G (2006). Lactobacillus vaginal microbiota of women attending a reproductive health care service in Benin city, Nigeria. Sex. Transm. Dis..

[36] Bourgeon L, Burke A, Higham T (2017). Earliest human presence in North America Dated to the Last Glacial Maximum: new radiocarbon dates from Bluefish Caves, Canada. PLoS ONE.

[37] Bortolini MC (2003). Y-chromosome evidence for differing ancient demographic histories in the Americas. Am. J. Hum. Genet..

[38] Hurtado, A. M., Hurtado, I. & Hill, K. Public health and adaptive immunity among natives of South America. *Lost Paradises and the Ethics of Research and Publication* 164–192 (Oxford University Press, New York, 2003).

[39] Lindenau J (2013). Distribution patterns of variability for 18 immune system genes in Amerindians–relationship with history and epidemiology. HLA.

[40] Bhatia KK, Black FL, Smith TA, Prasad ML, Koki GN (1995). Class I HLA antigens in two long-separated populations: Melanesians and South Amerinds. Am. J. Phys. Anthropol..

[41] Watkins DI (1992). New recombinant HLA-B alleles in a tribe of South American Amerindians indicate rapid evolution of MHC class I loci. Nature.

[42] Ewerton PD, de Meira Leite M, Magalhães M, Sena L, dos Santos EJM (2007). Amazonian Amerindians exhibit high variability of KIR profiles. Immunogenetics.

[43] Freire, G. & Tillett, A. *Salud indígena en Venezuela. First volume*. (Dirección de Salud Indígena, 2007).

[44] Contreras M (2010). The bacterial microbiota in the oral mucosa of rural Amerindians. Microbiology.

[45] Clemente JC (2015). The microbiome of uncontacted Amerindians. Sci. Adv..

[46] Blaser MJ (2012). Distinct cutaneous bacterial assemblages in a sampling of South American Amerindians and US residents. ISME J..

[47] Yatsunenko T (2012). Human gut microbiome viewed across age and geography. Nature.

[48] Godinho, N. M. D. O. *O Impacto das Migrações na Constituição Genética de Populações Latino-Americanas*. (2008).

[49] Jespers V (2015). The significance of *Lactobacillus crispatus* and *L. vaginalis* for vaginal health and the negative effect of recent sex: a cross-sectional descriptive study across groups of African women. BMC Infect. Dis..

[50] Gosmann C (2017). Lactobacillus-deficient cervicovaginal bacterial communities are associated with increased HIV acquisition in Young South African Women. Immunity.

[51] Godoy-Vitorino F (2018). Cervicovaginal fungi and bacteria associated with cervical intraepithelial neoplasia and high-risk Human Papillomavirus infections in a Hispanic population. Front. Microbiol..

[52] Tarnberg M, Jakobsson T, Jonasson J, Forsum U (2002). Identification of randomly selected colonies of lactobacilli from normal vaginal fluid by pyrosequencing of the 16S rDNA variable V1 and V3 regions. APMIS.

[53] Huse SM, Ye Y, Zhou Y, Fodor AA (2012). A core human microbiome as viewed through 16S rRNA sequence clusters. PLoS ONE.

[54] Shen J (2016). Effects of low dose estrogen therapy on the vaginal microbiomes of women with atrophic vaginitis. Sci. Rep..

[55] Witkin SS (2019). Vaginal biomarkers that predict cervical length and dominant bacteria in the vaginal microbiomes of pregnant women. mBio.

[56] Ravel J (2013). Daily temporal dynamics of vaginal microbiota before, during and after episodes of bacterial vaginosis. Microbiome.

[57] Smith BC (2012). The cervical microbiome over 7 years and a comparison of methodologies for its characterization. PLoS ONE.

[58] Gajer P (2012). Temporal dynamics of the human vaginal microbiota. Sci. Transl. Med..

[59] Kim TK (2009). Heterogeneity of vaginal microbial communities within individuals. J. Clin. Microbiol..

[60] Liu M-B (2013). Diverse vaginal microbiomes in reproductive-age women with vulvovaginal candidiasis. PLoS ONE.

[61] Verstraelen H (2009). Longitudinal analysis of the vaginal microflora in pregnancy suggests that *L. crispatus* promotes the stability of the normal vaginal microflora and that *L. gasseri* and/or *L. iners* are more conducive to the occurrence of abnormal vaginal microflora. BMC Microbiol..

[62] Byrne EH (2015). Cervicovaginal bacteria are a major modulator of host inflammatory responses in the female genital tract. Immunity.

[63] Mach N, Fuster-Botella D (2017). Endurance exercise and gut microbiota: a review. J. Sport Health Sci..

[64] Tasnim N, Abulizi N, Pither J, Hart MM, Gibson DL (2017). Linking the gut microbial ecosystem with the environment: does gut health depend on where we live?. Front. Microbiol..

[65] Collins MD (2006). The Prokaryotes.

[66] Freitas AC (2018). Increased richness and diversity of the vaginal microbiota and spontaneous preterm birth. Microbiome.

[67] Marrazzo JM, Thomas KK, Fiedler TL, Ringwood K, Fredricks DN (2008). Relationship of specific vaginal bacteria and bacterial vaginosis treatment failure in women who have sex with women. Ann. Intern. Med..

[68] Si J, You HJ, Yu J, Sung J, Ko G (2017). Prevotella as a hub for vaginal microbiota under the influence of host genetics and their association with obesity. Cell Host Microbe.

[69] Srinivasan S (2012). Bacterial communities in women with bacterial vaginosis: high resolution phylogenetic analyses reveal relationships of microbiota to clinical criteria. PLoS ONE.

[70] Yeoman CJ (2013). A multi-omic systems-based approach reveals metabolic markers of bacterial vaginosis and insight into the disease. PLoS ONE.

[71] Oakley BB, Fiedler TL, Marrazzo JM, Fredricks DN (2008). Diversity of human vaginal bacterial communities and associations with clinically defined bacterial vaginosis. Appl. Environ. Microbiol..

[72] Machado A, Cerca N (2015). Influence of biofilm formation by gardnerella vaginalis and other anaerobes on bacterial vaginosis. J. Infect. Dis..

[73] Vargas-Robles D (2018). High rate of infection by only oncogenic human papillomavirus in Amerindians. mSphere.

[74] Song D, Li H, Li H, Dai J (2015). Effect of human papillomavirus infection on the immune system and its role in the course of cervical cancer. Oncol. Lett..

[75] Briselden AM, Moncla BJ, Stevens CE, Hillier SL (1992). Sialidases (Neuraminidases) in bacterial vaginosis and bacterial vaginosis-associated microflora. J. Clin. Microbiol..

[76] Gillet E (2011). Bacterial vaginosis is associated with uterine cervical human papillomavirus infection: a meta-analysis. BMC Infect. Dis..

[77] Discacciati MG (2006). Presence of 20% or more clue cells: an accurate criterion for the diagnosis of bacterial vaginosis in Papanicolaou cervical smears. Diagn. Cytopathol..

[78] World Health Organization (2011). Haemoglobin Concentrations for the Diagnosis of Anaemia and Assessment of Severity.

[79] Magoč T, Salzberg SL (2011). FLASH: fast length adjustment of short reads to improve genome assemblies. Bioinformatics.

[80] Callahan BJ (2016). DADA2: High resolution sample inference from Illumina amplicon data. Nat. Methods.

[81] Allard G, Ryan FJ, Jeffery IB, Claesson MJ (2015). SPINGO: a rapid species-classifier for microbial amplicon sequences. BMC Bioinform..

[82] Cole JR (2013). Ribosomal Database Project: data and tools for high throughput rRNA analysis. Nucleic Acids Res..

[83] Morgulis A (2008). Database indexing for production MegaBLAST searches. Bioinformatics.

[84] Muzny CA (2013). Characterization of the vaginal microbiota among sexual risk behavior groups of women with bacterial vaginosis. PLoS ONE.

[85] Caporaso JG (2010). QIIME allows analysis of high-throughput community sequencing data. Nat. Methods.

[86] R: A language and environment for statistical computing (R Foundation for Statistical Computing, Vienna, Austria, 2016).

[87] Oksanen, J. *et al.* vegan: Community Ecology Package. R package version 2.4-4. *R Development Core Team. R: A language and environment for statistical computing. Vienna: R Foundation for Statistical Computing* (2010).

[88] Anderson MJ (2001). Permutation tests for univariate or multivariate analysis of variance and regression. Can. J. Fish. Aquat. Sci..

[89] Bates, D., Maechler, M., Bolker, B. & Walker, S. *Fitting Linear Mixed-Effects Models Using lme4*. (2015).

[90] textmineR: Functions for Text Mining and Topic Modeling v. 2.0.6 (2017).

[91] Rousseeuw PJ (1987). Silhouettes: a graphical aid to the interpretation and validation of cluster analysis. J. Comput. Appl. Math..

[92] Walther RTAG (2005). Cluster validation by prediction strength. J. Comput. Graph. Stat..

[93] Philentropy: Information Theory and Distance Quantification with R (2018).

[94] Hsieh TC, Ma KH, Chao A (2016). iNEXT: an R package for rarefaction and extrapolation of species diversity (Hill numbers). Methods Ecol Evol.

[95] Cohen J (1960). A coefficient of agreement for nominal scales. Educ. Psychol. Meas..

[96] Viera AJ, Garrett JM (2005). Understanding interobserver agreement: the kappa statistic. Fam. Med..

[97] Segata N (2011). Metagenomic biomarker discovery and explanation. Genome Biol..

[98] Nakazawa, M. fmsb: Functions for medical statistics book with some demographic data. *R package version 0.4* (2014).

[99] Benjamini Y, Hochberg Y (1995). Controlling the false discovery rate: a practical and powerful approach to multiple testing. J. R. Stat. Soc. B.

[100] Wickham H (2007). Reshaping Data with the reshape Package. J. Stat. Softw..

[101] gplots: Various R Programming Tools for Plotting Data (R, 2016).

[102] Vázquez-Baeza Y, Pirrung M, Gonzalez A, Knight R (2013). EMPeror: a tool for visualizing high-throughput microbial community data. Gigascience.

