# Supplementary material for: Changes in the vaginal microbiota across a gradient of urbanization
Source: Sci Rep. 2020 Jul 27;10:12487. doi: 10.1038/s41598-020-69111-x (PMC7385657; doi:10.1038/s41598-020-69111-x)
Supplement: Supplementary file 1 — Supplementary figures. [file 41598_2020_69111_MOESM1_ESM.pdf]

Supplementary figures

Changes in the vaginal microbiota across a gradient of urbanization

Daniela Vargas-Robles, Magda Magris, Natalia Morales, Iveth Rodríguez, Tahidid Nieves, Filipa Godoy-Vitorino, Luis David Alcaraz, María-Eglée Pérez, Jacques Ravel, Larry J. Forney, María Gloria Domínguez-Bello

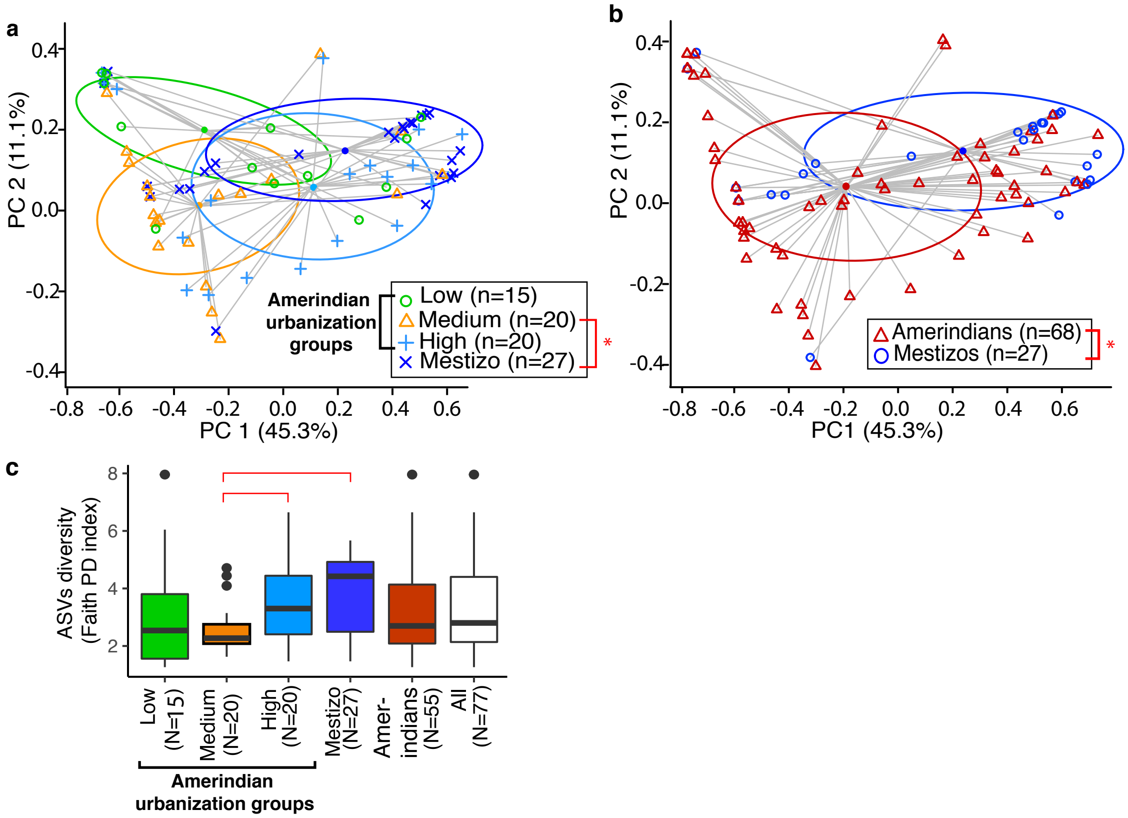

Fig S1. **Cervicovaginal microbiota diversity. a, b**, Principal Coordinate Analysis (PCoA) of unweighted UniFrac distance for all groups of women (**a**), and between ethnicities including all Amerindian women (**b**). Gray lines connect samples with the group centroid. Ellipses indicate one standard deviation. (**c**) Alpha diversity using Faith PD metric among all woman groups. Asterisks above red bars (*) connecting groups indicate significant differences even after p value adjustment for multiple comparisons; bars without asterisks indicate that significance is lost after p value adjustment.

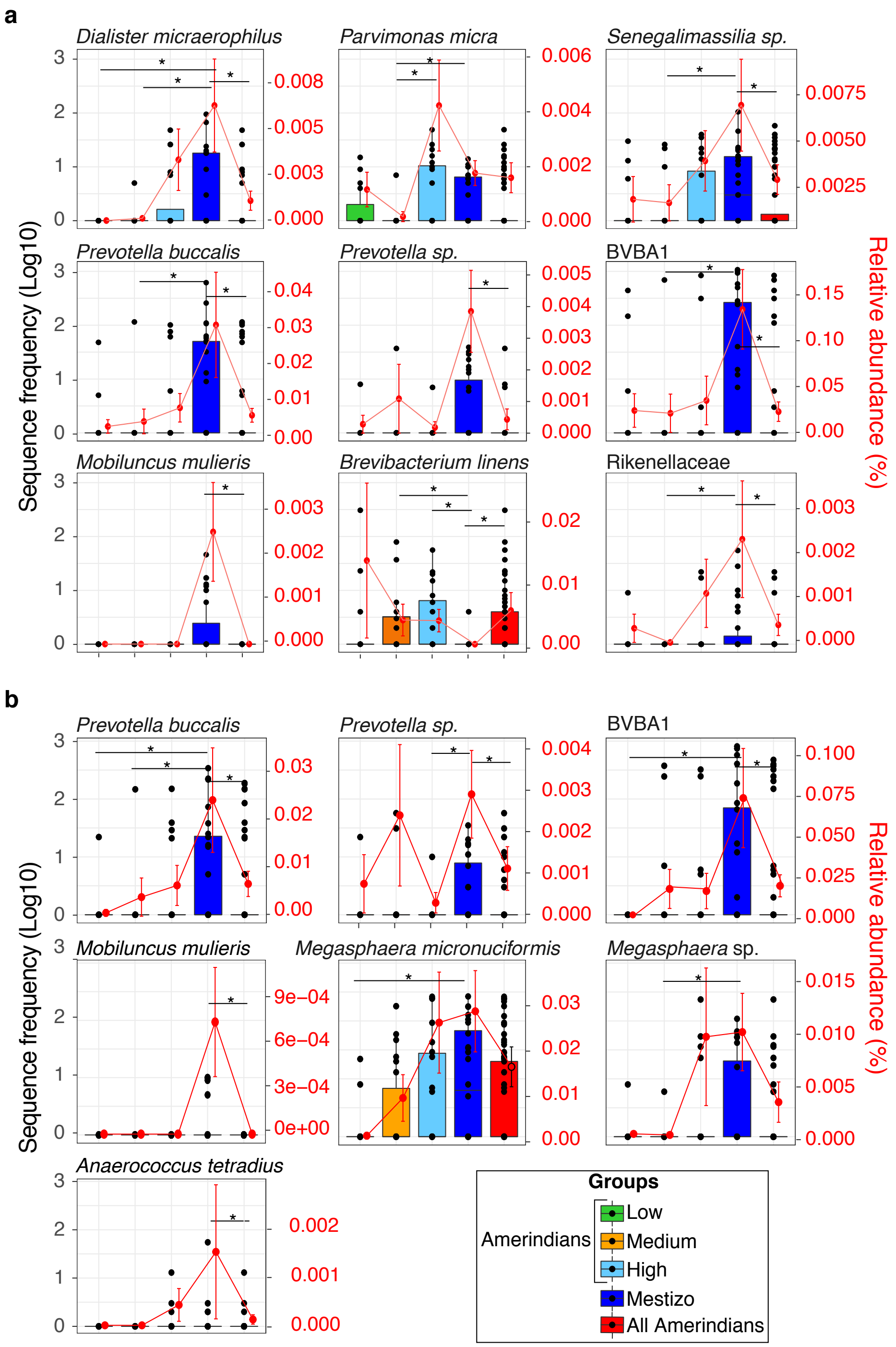

Fig. S2. Comparison among women groups for each LEfSe-based discriminant taxa. Sequence frequency (log-transformed) and relative abundance are shown in axes 'y' at the right and left of each plot respectably for cervicovaginal (**a**) and introital samples (**b**). Pair-wise comparison among all for groups and between ethnicities (all Amerindians *vs*. mestizos) were performed with Kruskal-Wallis test (asterisk indicate significant differences between groups, p<0.050).

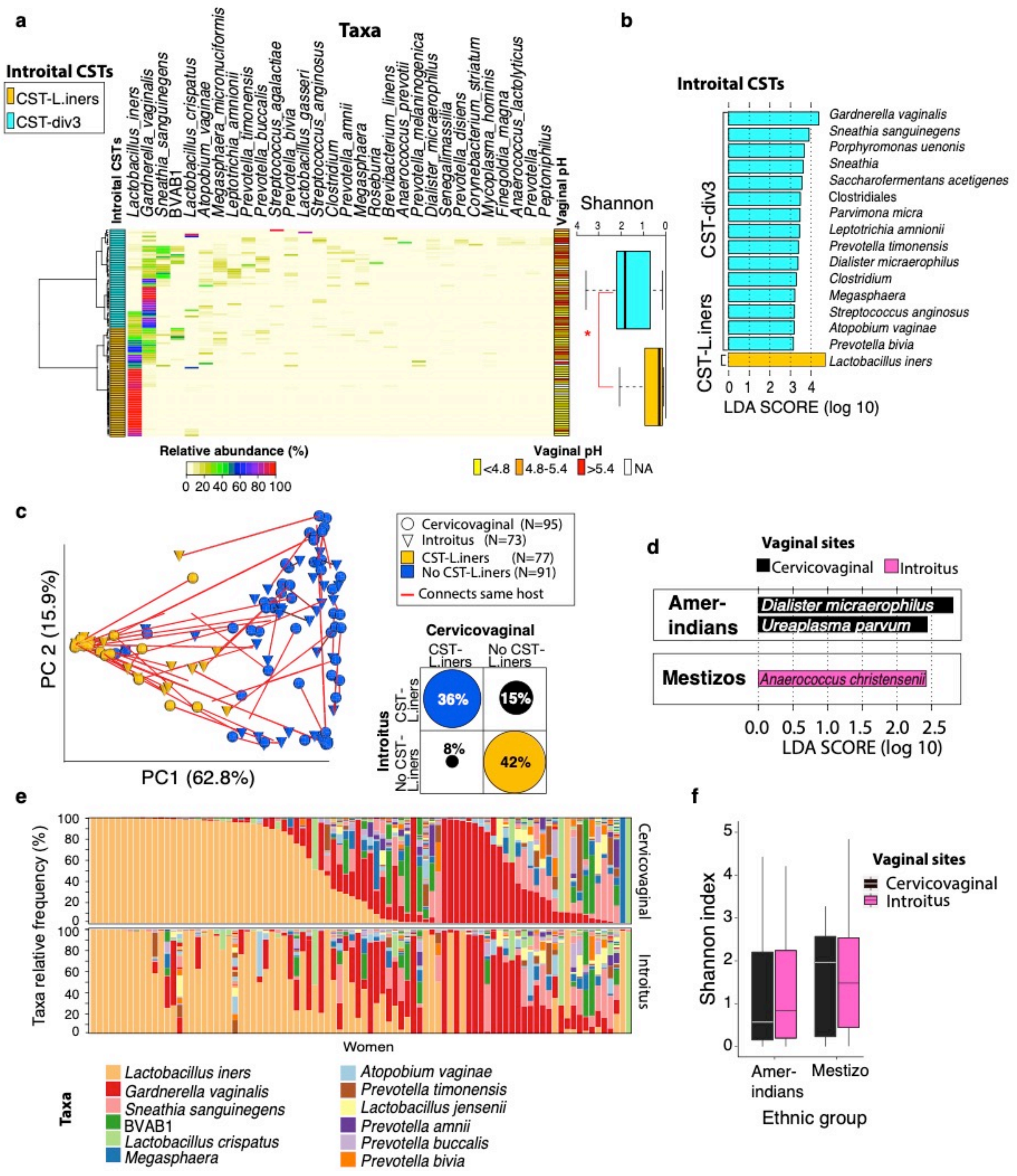

Fig. S3. Cervicovaginal and introital microbiota diversity. a, Heatmap with hierarchical clustering of women showing the 30 most abundant introital taxa. Clustering yield two introital community state types (CSTs) labeled as CST-L.iners (dominated by *Lactobacillus iners*) and CST-div3 (diverse bacterial assembly). Boxplot based on Shannon index for each introital CST showed significant differences marked with asterisks (p.adj<0.001, Kruskal-Wallis). **b**, Discriminant taxa analysis for introital CSTs according to LEfSe, p<0.010. **c,** Principal Coordinate Analysis (PCoA) for weighted UniFrac distances by body

site (symbols) and CSTs (CST-L. iners or no CST-*L.iners* (colors)). Red lines connect introital and cervicovaginal samples from the same woman. No statistical differences were observed between body sites (p=0.243, R^2=0.011, PERMANOVA). There was a 78% of total coincidence between body sites from the same woman (Cohen's Kappa: 0.553, "moderate" strength of agreement). **d**, Discriminant taxa analysis (p<0.010, LEfSe) within Amerindians or mestizos by body site. **e**, Summary for all cervicovaginal (top) and introital (bottom) taxa. Cervicovaginal samples were sorted by *L. iners* and *G. vaginalis* relative abundance. Color legend shows the 12 most abundant taxa. **f**, Microbial Shannon diversity vaginal site within Amerindians or mestizos.

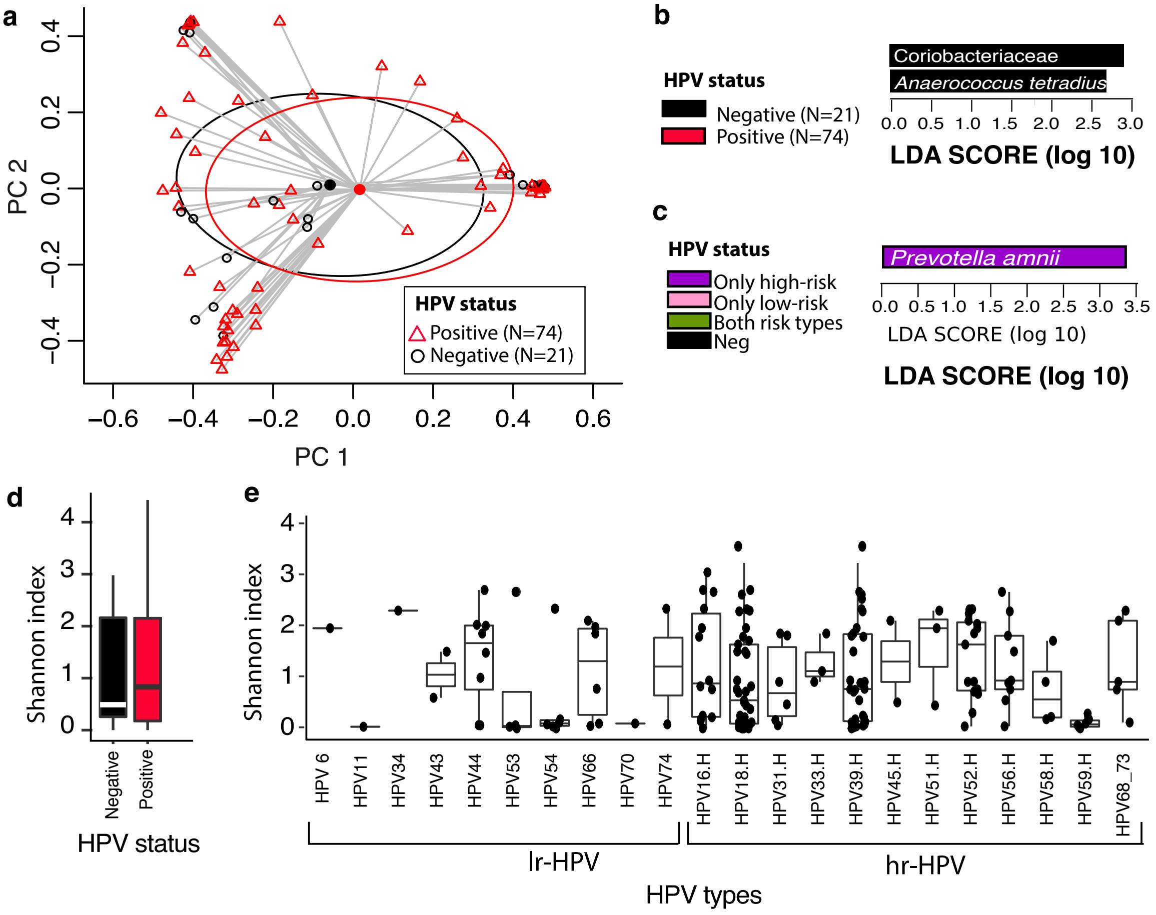

Fig. S4. Cervicovaginal beta, alpha microbial diversities and discriminant taxa by HPV status by urbanization groups and ethnicity. a, Principal Coordinate Analysis (PCoA) for Bray-Curtis dissimilarity. Gray lines connect samples with the group centroid. Ellipses indicate one standard deviation. No significant differences were detected. **b, c,** Discriminant taxa analysis according to LEfSe ($p<0.010$) by HPV status showing that only HPV negative women **(b)** and only infected with high-risk HPV **(c)** yield discriminant-taxa. **d,** Shannon diversity by HPV status did not differ ($p>0.050$, Kruskal-Wallis test). **f**, Shannon diversity by HPV type did not differ ($p>0.050$, Kruskal-Wallis test).
