# Supplementary material for: Changes in the vaginal microbiota across a gradient of urbanization
Source: Sci Rep. 2020 Jul 27;10:12487. doi: 10.1038/s41598-020-69111-x (PMC7385657; doi:10.1038/s41598-020-69111-x)
Supplement: Supplementary file 2 — Supplementary tables. [file 41598_2020_69111_MOESM2_ESM.pdf]

Supplementary tables

Changes in the vaginal microbiota across a gradient of urbanization

Daniela Vargas-Robles, Magda Magris, Natalia Morales, Iveth Rodríguez, Tahidid Nieves, Filipa Godoy-Vitorino, Luis David Alcaraz, María-Eglée Pérez, Jacques Ravel, Larry J. Forney, María Gloria Domínguez-Bello

Table S1. Lifestyle variables of 111 women by urbanization level and ethnicity. Red values indicate p<0.05 or p.adj<0.05.

	Prevalence % (n/N)				*p-value (p.adj)****	
	**Amerindians by urbanization groups (N=67[&])**					
**Variables**	**Low (N=22)**	**Medium (N=22)**	**High (N=23)**	**Mestizos (N=29)**	***Amerindian urbanization groups***	***Ethnicity***
**Diet last 24 hours***					*$1x10^{-12}$ ($1x10^{-11}$)*	*0.416*
**Taditional**	81.8 (18/22)	13.6 (3/22)	0 (0/23)	0.0 (0/24)		
**Mixed**	9.1 (2/22)	63.6 (14/22)	17.4 (4/23)	8.3 (2/24)		
**Industrialized or non traditional diet**	9.1 (2/22)	22.7 (5/22)	82.6 (19/23)	91.7 (22/24)		
**Finish elementary school**	31.8 (7/22)	86.4 (19/22)	95.7 (22/23)	95.8 (23/24)	*$2x10^{-6}$ ($1x10^{-5}$)*	*1.000*
**Practice crop gardening**	90.9 (20/22)	90.9 (20/22)	52.2 (12/23)	4.2 (1/24)	*0.003 (0.009)*	*$2x10^{-4}$ (0.002)*
**History of sexual contact with Mestizo**	0.0 (0/22)	22.7 (5/22)	34.8 (8/23)	100 (29/29)	*0.006 (0.014)*	*$1x10^{-7}$ ($1x10^{-6}$)*
**Number of pregnancies, mean [min, max]**	5.9 [0-12]	5.0 [0-12]	2.3 [0-8]	2.3 [0-11]	*0.003 (0.009)*	*0.862*
**Use vaginal douche**	0.0 (0/22)	0.0 (0/22)	0.0 (0/22)	41.4 (12/29)	*NA*	*$4x10^{-4}$ (0.002)*
**Sexual contact type**					*0.660*	*0.006 (0.023)*
**Vaginal**	100 (22/22)	95.5 (21/22)	100 (23/23)	72.4 (21/29)		
**Vaginal + anal**	0.0 (0/22)	4.5 (1/22)	0.0 (0/23)	0.0 (0/29)		
**Vaginal + oral**	0.0 (0/22)	0.0 (0/22)	0.0 (0/23)	27.6 (8/29)		
**Time of the last antibiotic consumption****					*0.152*	*0.076*
**≤ 4 months**	0 (0/22)	9.1 (2/22)	18.2 (4/22)	42.9 (12/28)		
**> 4 months**	100 (22/22)	90.9 (20/22)	81.8 (18/22)	57.1 (16/28)		
**Hormonal contraception use**	4.5 (1/22)	0.0 (0/22)	4.5 (1/22)	25.9 (7/27)	*NA*	*0.059*
**Patient was born by c-section**	0.0 (0/21)	0.0 (0/20)	0.0 (0/20)	10.7 (3/28)	*NA*	*0.242*
**Smoking currently**	0.0 (0/22)	4.5 (1/22)	8.7 (2/23)	13.8 (4/29)	*0.768*	*0.682*
**Breastfeeding currently**	76.2 (16/21)	50.0 (11/22)	40.9 (9/22)	74.1 (20/27)	*0.056*	*0.023 (0.070)*
**Number of sexual partner last 60 days [&&]**					*0.602*	*0.107*
**None**	22.7 (5/22)	22.7 (5/22)	34.8 (8/23)	14.3 (4/28)		
**One**	77.3 (17/22)	77.3 (17/22)	65.2 (15/23)	85.7 (24/28)		
**Abortions**					*0.346*	*0.529*
**None**	52.6 (10/19)	65.0 (13/20)	75.0 (15/20)	63.0 (17/27)		
**One or more**	47.4 (9/19)	35.0 (7/20)	25.0 (5/20)	37.0 (10/27)		
**Surgical intervention history**	81.8 (18/22)	90.9 (20/22)	95.7 (22/23)	89.7 (26/29)	*0.287*	*0.621*

*Traditional diet: eat at least 80% of exclusively traditional food (yucca -cassava, manioc- other tubers, insects, forest fruits and huntting meat. Mixed diet: eat more than 20% of indistrilized food and at least one item classified as exclusively traditional. Industrialized or non traditional diet: eat 80% or more of industrilized food (market food or from farms including, corn flour, wheat flour, rice, chicken, grains, red meat, cow milk, hen eggs, cheese, processed sauses, soda, food in cans, pork, yogurt and snaks) and non of the exclusively traditional food.

**No women took any antibiotic a month pre sampling

***Fisher´s exact test or ANOVA were applied. Adjusted p-values for multiple comparisons were performed using false discovery rate Benjamini-Hochberg (fdr-BH) method.

& Differences of N in "Amerindians by urbanization groups" vs. "All Amerindians" is due to missing urbanization level information necessary for grouping them from some women.

&& The "more than one category" was included although no value was registered here.

NA cells indicate that statistical test could not be performed due to small n per category

Table S2. Health variables of 111 women by urbanization level and ethnicity. Red values indicate p<0.05 or p.adj<0.05.

Variables	Amerindians by urbanization groups (N=67[&])			Mestizo (N=29)	*p-value (p adjusted [&&])*	
	Low (N=22)	Medium (N=22)	High (N=23)		Amerindian urbanization groups	*Ethnicity*
**Body Mass Index *, mean (sd)**	22.1 (1.9)	24.8 (4.8)	26.2 (4.5)	27.2 (5.9)	*0.004 (0.048)*	0.505
**Woman reporting vaginal discomfort and/or discharges**	18.2 (4/22)	59.1 (13/22)	26.1 (6/23)	20.7 (6/29)	*0.013 (0.078)*	*0.745*
**Intestinal helminthes prevalence (%)**	75.0 (15/20)	65.0 (13/20)	33.3 (5/15)	28.6 (2/7)	*0.043 (0.172)*	*1.000*
**Hemoglobine level, mean (sd)**	12.7 (1.3)	12.7 (1.4)	12.9 (1.1)	13.7 (0.9)	*0.909*	*0.014 (0.167)*
**Age, mean (sd)**	31.1 (9.5)	31.2 (7.8)	29.2 (8.6)	26.6 (9.6)	*0.682*	*0.327*
**Anemia** (%)**	27.3 (6/22)	27.3 (6/22)	13.0 (3/23)	3.4 (1/29)	*0.387*	*0.109*
**Vaginal pH**					*0.899*	*0.322*
4-4.7	47.3 (9/19)	47.6 (10/21)	54.5 (12/22)	50.0 (13/26)		
4.8-5.4	26.3 (5/19)	33.3 (7/21)	31.8 (7/22)	46.2 (12/26)		
5.5-7	26.3 (5/19)	19.0 (4/21)	13.6(3/22)	3.9 (1/26)		
**Cervical HPV prevalence (%)**	63.6 (14/22)	68.2 (15/22)	78.3 (18/23)	82.8 (24/29)	*0.527*	*0.734*
**Cervical High risk-HPV*** prevalence (%)**	54.5 (12/22)	68.2 (15/22)	78.3 (18/23)	65.5 (19/29)	*0.243*	*0.368*
**Cervical abnormalities prevalence (%)**	9.1 (2/22)	18.2 (4/22)	4.3 (1/23)	0.0 (0/29)	*0.287*	*0.442*
**Menstrual cycle phase**					*0.570*	*0.372*
**Not bleeding (Breastfeeding)**	53.8 (7/13)	35 (7/20)	22.2 (4/18)	16.7 (4/24)		
**Follicular**	23.1 (3/13)	20 (4/20)	16.7 (3/18)	41.7 (10/24)		
**Luteal**	15.4 (2/13)	35 (7/20)	44.4 (8/18)	33.3 (8/24)		
**Periovulatory**	7.7 (1/13)	10 (2/20)	16.7 (3/18)	8.3 (2/24)		
**Cytological cells with ≥20% of clue cells**	4.5 (1/22)	9.1 (2/22)	21.7 (5/23)	20.7 (6/29)	*0.252*	*1.000*

* According to WHO: Underweight: below 18.5, Normal weight: 18.5–24.9, Pre-obesity: 25.0–29.9, Obesity class I: 30.0–34.9, Obesity class II: 35.0–39.9, Obesity class III: above 40.

**Anemia is defined by the WHO as the hemoglobin levels lower than 12 mg/L

*** High risk-HPV types detected by the LiPA25 test:16, 18, 31, 33, 35, 39, 45, 51, 52, 56, 58, 59.

& Differences of N in "Amerindians by urbanization groups" vs. "All Amerindians" is due to missing urbanization level information from some women necessary for grouping them.

&& Adjusted p-values for multiple comparisons were performed using false discovery rate Benjamini-Hochberg (fdr-BH) method, appltying ANOVA, Kruskal-Wallis and Fisher´s exact test depending on the data tipe and distribution

Table S3. Cervicovaginal and introital unrarefied and rarefied 16S rRNA gene sequences and number of different taxa from Amplicon Sequence Variants (ASVs) summary. Sum, mean and standard deviation (sd) of sequences and taxa from ASVs is shown. Rarefaction was performed at 1,655 sequences/sample

		Parameters		***Total***	Controls **
		**N samples**		***111***	**8**
Introital	Unrarefied	Sequences yielding ASVs	Sum	***1,707,270***	153
			Mean	***15,521***	19
			Sd	***14,974***	7
		Observed ASVs	Sum	**806**	6
			Mean	**20.6**	1
			Sd	**18.5**	0
		Observed ASV taxa	Sum	***187***	3***
			Mean	***15.5***	0.4
			Sd	***10.8***	0
	Rarefied	**N samples**		***101***	-
		Sequences yielding ASVs	Sum	***167,155***	-
			Mean	***1,655***	-
			Sd	***0.0***	-
		Observed ASVs	Sum	**727**	
			Mean	**20.1**	
			Sd	**17.3**	
		Observed ASV taxa	Sum	***161***	-
			Mean	***11.8***	-
			Sd	***9.9***	-
Cervicovaginal	Unrarefied	Sequences yielding ASVs	Sum	***1,063,897***	-
			Mean	***9,672***	-
			Sd	***10,003***	-
		Observed ASVs	Sum	**580**	
			Mean	**15.1**	
			Sd	**13.6**	
		Observed ASV taxa	Sum	***128***	-
			Mean	***9.2***	-
			Sd	***8.0***	-
	Rarefied	**N samples**		***95***	-
		Sequences yielding ASVs	Sum	***157,225***	-
			Mean	***1,655***	-
			Sd	***0.0***	-
		Observed ASVs	Sum	**521**	
			Mean	***15.4***	
			Sd	***13.6***	
		Observed ASV taxa	Sum	***115***	-
			Mean	***9.2***	-
			Sd	***7.8***	-

* Differences of N in "Amerindians by urbanization groups" vs. "All Amerindians" is due to missing urbanization level information from some women necessary for grouping them.

**Environmental controls taken by exposuring a swabs 30s in the air

****Lactobacillus iners, Brevibacterium linens, Megasphaera*

Table S4. Megablast against NCBI database for the sequences that failed taxa assignment.

Description	Query cover	E value	Identity	Accession	Isolation source	N sequences	N of occurrence (N women)	Host ID	Women group*	Body site
Uncultured bacterium clone	0.85	1E-151	0.92	JX871228.1	vagina	29	1	30	Amerindian	cervicovaginal site
Uncultured bacterium clone	0.97	0	0.93	JX871293.1	vagina	32	2	45, 30	Mestizo, Amerindian	introitus, introitus
Uncultured bacterium clone	0.85	4E-165	0.94	JX871228.1	vagina	42	1	30	Amerindian	cervicovaginal site
Uncultured bacterium clone	0.85	4E-175	0.95	JX871228.1	vagina	16	1	45	Mestizo	cervicovaginal site
Uncultured bacterium clone	0.85	4E-175	0.95	JX871228.1	vagina	41	2	50, 30	Amerindian, Amerindian	cervicovaginal site, introitus
Uncultured bacterium clone	0.85	1E-176	0.96	JX871228.1	vagina	72	2	3, 3	Mestizo, Mestizo	introitus, cervicovaginal site
Uncultured bacterium clone	0.85	1E-176	0.96	JX871228.1	vagina	135	2	97, 97	Low, Low	introitus, cervicovaginal site
Uncultured bacterium clone	0.85	1E-175	0.96	JX871228.1	vagina	22	2	107, 112	Amerindian, Low	introitus, cervicovaginal site
Uncultured Lachnospiraceae	0.95	0	0.97	KF506154.1	skin	426	2	15, 15	High, High	introitus, cervicovaginal site
Uncultured bacterium clone	0.87	0	0.97	JX871228.1	vagina	43	1	21	High	cervicovaginal site
Gardnerella vaginalis HMP9231, complete	1	0	0.97	CP002725.1	NA	11	1	116	High	cervicovaginal site
Uncultured bacterium clone	0.81	0	0.99	JX871228.1	vagina	42	1	3	Mestizo	introitus
Uncultured bacterium clone	0.79	0	0.99	JX871228.1	vagina	17	1	30	Amerindian	introitus
Uncultured Uncultured Bacteroidales bacterium	1	0	1	LT692716.1	vagina	41	1	3	Mestizo	cervicovaginal site
Uncultured bacterium clone	0.8	0	1	GU098717.1	human colon	11	1	30	Amerindian	introitus
Uncultured bacterium clone	0.85	0	1	JX871228.1	vagina	78	1	42	Mestizo	introitus
Uncultured bacterium clone	0.85	0	1	JX871228.1	vagina	82	2	51, 21	Mestizo, High	cervicovaginal site, introitus
Uncultured bacterium clone	1	0	1	HM324642.1	skin, popliteal	13	1	82	Amerindian	cervicovaginal site

* Cells indicating "Amerindians" correspond to Amerindian women from wich urbanization level was not possible to obtain.

Table S5. Cervicovaginal and introital microbiota alpha diversity metrics by urbanization group and ethnicity. Red values indicate p<0.05 or p.adj<0.05.

Diversity metrics	Comparison groups	*p-value*	
		Cervix *	Introitus **
Shannon	Amerindian urbanization groups	0.078	0.357
	Ethnicity&&&	0.844	0.771
Simpson	Amerindian urbanization groups&&	0.061	0.297
	Ethnicity	0.973	0.923
Bray Curtis dissimilarity (PERMANOVA)	Amerindian urbanization groups&&	0.340 (R^2=0.056)	0.415 (R^2=0.037)
	Ethnicity	0.384 (R^2=0.027)	0.322 (R^2=0.028)

* For Amerindian urbanization groups: low, N=15; medium, N=20; high, N=20. For ethnicity Ameridians, N=15; mestizo, N=27

** For Amerindian urbanization groups: low, N=19; medium, N=22; high, N=22. For ethnicity Ameridians, N=17; mestizo, N=24

Table S6. Taxa relative abundance by urbanization level and ethnicity by body site. Cells are colored in a gradient from lower (blue) to higher (red) values.

	Relative abundance of each taxa per group (%)									
	**Cervicovaginal site**					**Introitus**				
	Amerindian urbanization groups					Amerindian urbanization groups				
Taxa	Low	Medium	High	Mestizos	**Total**	Low	Medium	High	Mestizos	**Total**
N women	15	20	20	27	**95**	18	22	23	24	**101**
Actinomyces turicensis	0.06	0.00	0.00	0.00	**0.01**	0.15	0.00	0.00	0.01	**0.04**
Aerococcus christensenii	0.01	0.00	0.20	0.02	**0.09**	0.00	0.02	0.43	0.03	**0.13**
Aggregatibacter aphrophilus	0.00	0.00	0.00	0.00	**0.00**	0.00	0.05	0.00	0.00	**0.01**
Alloprevotella rava	0.09	0.00	0.00	0.00	**0.01**	0.00	0.00	0.00	0.00	**0.01**
Alloscardovia omnicolens	0.00	0.00	0.00	0.00	**0.00**	0.00	0.00	0.00	0.00	**0.00**
Anaerococcus	0.00	0.00	0.00	0.00	**0.00**	0.11	0.00	0.00	0.00	**0.02**
Anaerococcus hydrogenalis	0.00	0.00	0.00	0.00	**0.00**	0.10	0.01	0.00	0.33	**0.10**
Anaerococcus lactolyticus	0.07	0.00	0.00	0.00	**0.01**	0.70	0.01	0.02	0.20	**0.18**
Anaerococcus murdochii	0.02	0.00	0.00	0.00	**0.00**	0.03	0.00	0.00	0.00	**0.01**
Anaerococcus obesiensis	0.08	0.00	0.00	0.00	**0.01**	0.07	0.00	0.00	0.03	**0.03**
Anaerococcus octavius	0.00	0.00	0.00	0.00	**0.00**	0.02	0.02	0.00	0.02	**0.01**
Anaerococcus prevotii	0.03	0.00	0.00	0.00	**0.01**	2.39	0.00	0.02	0.00	**0.42**
Anaerococcus tetradius	0.00	0.00	0.05	0.00	**0.01**	0.00	0.00	0.04	0.13	**0.04**
Anaerococcus vaginalis	0.00	0.00	0.00	0.00	**0.00**	0.00	0.05	0.00	0.00	**0.01**
Arcanobacterium	0.00	0.00	0.00	0.00	**0.00**	0.00	0.00	0.00	0.00	**0.00**
Arcanobacterium phocae	0.00	0.00	0.00	0.00	**0.00**	0.00	0.00	0.00	0.00	**0.00**
Arthrobacter albus	0.00	0.00	0.00	0.00	**0.00**	0.00	0.00	0.00	0.00	**0.00**
Arthrobacter cumminsii	0.00	0.00	0.00	0.00	**0.00**	0.00	0.00	0.00	0.00	**0.00**
Atopobium parvulum	0.00	0.00	0.00	0.00	**0.00**	0.00	0.00	0.00	0.00	**0.00**
Atopobium vaginae	0.46	0.96	3.49	0.54	**1.57**	0.98	2.93	4.06	0.72	**2.31**
Bacteroidales	0.00	0.01	0.04	0.01	**0.01**	0.00	0.01	0.03	0.03	**0.03**
Bacteroides coagulans	0.18	0.00	0.00	0.00	**0.03**	0.02	0.00	0.01	0.00	**0.02**
Bacteroides uniformis	0.00	0.00	0.00	0.00	**0.00**	0.00	0.00	0.01	0.00	**0.00**
Bifidobacterium bifidum	0.00	0.00	0.00	0.00	**0.00**	0.00	0.01	0.00	0.02	**0.01**
Bifidobacterium breve	0.00	0.00	0.14	0.00	**0.03**	0.00	0.00	0.07	0.00	**0.01**
Bifidobacterium longum	0.00	0.00	0.01	0.01	**0.01**	0.00	0.02	0.03	0.01	**0.01**
Brachybacterium alimentarium	0.08	0.02	0.04	0.00	**0.02**	0.03	0.01	0.00	0.01	**0.02**
Brachybacterium tyrofermentans	0.05	0.01	0.02	0.00	**0.01**	0.00	0.04	0.01	0.02	**0.02**
Brevibacterium	0.01	0.01	0.00	0.00	**0.00**	0.00	0.00	0.01	0.00	**0.00**
Brevibacterium linens	1.35	0.37	0.39	0.00	**0.37**	0.32	0.48	0.22	0.30	**0.45**
Brevibacterium massiliense	0.01	0.00	0.00	0.00	**0.00**	0.00	0.00	0.00	0.00	**0.00**
Bulleidia extructa	0.00	0.00	0.00	0.00	**0.00**	0.00	0.00	0.01	0.00	**0.00**
Campylobacter	0.00	0.00	0.00	0.00	**0.00**	0.00	0.00	0.00	0.00	**0.00**
Campylobacter faecalis	0.00	0.00	0.01	0.00	**0.00**	0.00	0.02	0.01	0.01	**0.01**
Campylobacter ureolyticus	0.16	0.00	0.03	0.00	**0.03**	0.38	0.00	0.05	0.12	**0.13**
Clostridiales	0.09	0.01	0.00	0.02	**0.20**	0.01	0.34	0.02	0.01	**0.09**
Clostridium	0.03	0.37	0.62	0.44	**0.40**	0.01	0.73	1.48	0.51	**0.62**
Clostridium perfringens	0.00	0.00	0.00	0.01	**0.00**	0.00	0.00	0.00	0.00	**0.00**
Clostridium stercorarium	0.00	0.07	0.00	0.00	**0.01**	0.00	0.05	0.01	0.01	**0.02**
Comamonadaceae	0.00	0.00	0.00	0.00	**0.00**	0.00	0.00	0.00	0.00	**0.00**
Coriobacteriaceae	0.00	0.00	0.08	0.00	**0.02**	0.00	0.03	0.03	0.01	**0.01**
Corynebacteriaceae	0.00	0.00	0.00	0.00	**0.00**	0.00	0.01	0.00	0.04	**0.01**
Corynebacterium	0.10	0.00	0.00	0.00	**0.02**	0.02	0.00	0.02	0.01	**0.11**
Corynebacterium afermentans	0.00	0.00	0.00	0.00	**0.00**	0.00	0.00	0.00	0.00	**0.00**
Corynebacterium amycolatum	0.15	0.00	0.00	0.00	**0.02**	0.03	0.04	0.04	0.04	**0.09**
Corynebacterium atypicum	0.00	0.00	0.00	0.00	**0.00**	0.00	0.01	0.00	0.00	**0.00**
Corynebacterium aurimucosum	0.09	0.00	0.00	0.00	**0.01**	0.00	0.00	0.01	0.15	**0.09**
Corynebacterium coyleae	0.00	0.00	0.00	0.00	**0.00**	0.00	0.00	0.00	0.00	**0.01**
Corynebacterium freneyi	0.00	0.03	0.00	0.00	**0.01**	0.00	0.01	0.00	0.00	**0.00**
Corynebacterium glucuronolyticum	0.00	0.00	0.00	0.00	**0.01**	0.00	0.00	0.00	0.00	**0.00**
Corynebacterium jeikeium	0.01	0.00	0.00	0.00	**0.00**	0.00	0.00	0.00	0.00	**0.02**
Corynebacterium pseudogenitalium	0.00	0.00	0.00	0.00	**0.00**	0.04	0.00	0.00	0.03	**0.02**

Corynebacterium pyruviciproducens	0.04	0.00	0.00	0.00	**0.01**	0.30	0.01	0.00	0.00	**0.08**
Corynebacterium riegelii	0.00	0.00	0.00	0.00	**0.00**	0.00	0.00	0.00	0.00	**0.00**
Corynebacterium simulans	0.00	0.00	0.00	0.00	**0.00**	0.00	0.01	0.00	0.07	**0.02**
Corynebacterium striatum	0.00	0.00	0.00	0.00	**0.00**	0.00	0.03	0.00	0.75	**0.18**
Corynebacterium sundsvallense	0.00	0.00	0.00	0.00	**0.00**	0.00	0.00	0.00	0.00	**0.00**
Corynebacterium tuscaniense	0.00	0.00	0.00	0.00	**0.00**	0.00	0.00	0.00	0.00	**0.00**
Corynebacterium urealyticum	0.00	0.00	0.00	0.00	**0.00**	0.00	0.00	0.00	0.00	**0.00**
Cystobacter	0.00	0.00	0.00	0.00	**0.00**	0.00	0.00	0.00	0.00	**0.01**
Dermabacter hominis	0.00	0.00	0.00	0.00	**0.00**	0.00	0.00	0.00	0.00	**0.00**
Dialister	0.00	0.00	0.01	0.06	**0.02**	0.03	0.00	0.00	0.01	**0.01**
Dialister micraerophilus	0.00	0.00	0.40	0.45	**0.27**	0.38	0.26	0.18	0.35	**0.30**
Dialister propionicifaciens	0.13	0.00	0.00	0.00	**0.02**	0.00	0.00	0.00	0.04	**0.01**
Dietzia	0.09	0.06	0.06	0.00	**0.04**	0.04	0.06	0.04	0.04	**0.05**
Dorea longicatena	0.00	0.00	0.00	0.00	**0.00**	0.00	0.00	0.00	0.00	**0.00**
Escherichia/Shigella	0.00	0.00	0.00	0.00	**0.00**	0.00	0.01	0.00	0.00	**0.00**
Eubacterium oxidoreducens	0.00	0.00	0.00	0.00	**0.00**	0.00	0.00	0.01	0.00	**0.00**
Eubacterium rectale	0.00	0.00	0.00	0.00	**0.00**	0.00	0.00	0.02	0.00	**0.00**
Eubacterium yurii	0.02	0.00	0.01	0.00	**0.00**	0.00	0.00	0.00	0.01	**0.00**
Ezakiella	0.06	0.00	0.00	0.00	**0.01**	0.00	0.00	0.00	0.00	**0.00**
Facklamia hominis	0.00	0.00	0.00	0.00	**0.00**	0.00	0.00	0.00	0.00	**0.00**
Faecalibacterium prausnitzii	0.00	0.00	0.00	0.00	**0.00**	0.00	0.00	0.01	0.00	**0.00**
Fastidiosipila	0.00	0.16	0.10	0.02	**0.07**	0.00	0.35	0.07	0.02	**0.09**
Finegoldia magna	0.07	0.00	0.00	0.00	**0.02**	0.36	0.05	0.03	0.19	**0.18**
Fusobacterium	0.00	0.00	0.04	0.00	**0.01**	0.00	0.00	0.00	0.00	**0.00**
Fusobacterium gonidiaformans	0.00	0.00	0.08	0.00	**0.02**	0.00	0.00	0.00	0.00	**0.00**
Fusobacterium naviforme	0.00	0.00	0.09	0.00	**0.02**	0.00	0.00	0.19	0.03	**0.05**
Fusobacterium nucleatum	0.00	0.00	0.00	0.28	**0.08**	0.00	0.00	0.00	0.02	**0.01**
Gardnerella vaginalis	12.02	25.29	32.61	14.47	**22.38**	20.65	25.94	31.86	19.29	**26.27**
Gemella asaccharolytica	0.00	0.02	0.06	0.00	**0.03**	0.02	0.04	0.08	0.01	**0.04**
Gemella haemolysans	0.03	0.00	0.00	0.00	**0.01**	0.00	0.00	0.00	0.00	**0.00**
Gordonibacter pamelaeae	0.00	0.00	0.00	0.00	**0.00**	0.02	0.00	0.00	0.00	**0.00**
Granulicatella elegans	0.23	0.00	0.01	0.00	**0.04**	0.17	0.00	0.03	0.00	**0.09**
Haemophilus haemolyticus	0.07	0.00	0.00	0.00	**0.01**	0.02	0.00	0.00	0.00	**0.00**
Haemophilus parainfluenzae	0.00	0.00	0.00	0.00	**0.00**	0.00	0.00	0.00	0.01	**0.00**
Helcococcus sueciensis	0.00	0.00	0.00	0.00	**0.00**	0.00	0.00	0.00	0.00	**0.00**
Howardella ureilytica	0.00	0.00	0.00	0.00	**0.00**	0.00	0.00	0.00	0.01	**0.00**
Lachnospira	0.02	0.00	0.02	0.00	**0.01**	0.00	0.01	0.00	0.01	**0.00**
Lachnospiraceae	0.00	0.00	0.00	0.00	**0.00**	0.00	0.01	0.00	0.00	**0.00**
Lactobacillus crispatus	0.60	12.47	2.68	0.48	**3.29**	0.18	9.24	2.47	1.26	**3.00**
Lactobacillus equicursoris	0.00	0.02	0.00	0.00	**0.00**	0.00	0.00	0.00	0.00	**0.00**
Lactobacillus gasseri	0.00	4.69	0.00	0.00	**0.95**	0.00	4.08	0.03	0.00	**0.86**
Lactobacillus iners	55.84	44.31	21.77	44.16	**40.98**	60.92	40.34	37.59	50.04	**43.07**
Lactobacillus jensenii	0.08	0.05	0.00	0.21	**0.08**	0.00	0.18	0.00	0.22	**0.09**
Lactobacillus vaginalis	0.00	0.00	0.00	0.00	**0.00**	0.00	0.00	0.00	0.01	**0.00**
Leptotrichia amnionii	1.30	2.76	0.74	2.32	**1.70**	0.92	2.06	0.36	1.98	**1.16**
Leucobacter komagatae	0.00	0.00	0.00	0.00	**0.00**	0.00	0.00	0.00	0.00	**0.00**
Megasphaera	0.24	0.02	0.96	1.05	**0.75**	0.02	0.01	0.90	0.80	**0.48**
Megasphaera micronuciformis	1.54	0.73	2.85	4.48	**3.18**	0.08	0.90	2.68	2.67	**1.90**
Megasphaera sueciensis	0.00	0.00	0.00	0.00	**0.00**	0.00	0.00	0.00	0.00	**0.00**
Mobiluncus curtisii	0.04	0.00	0.04	0.01	**0.02**	0.00	0.00	0.12	0.00	**0.02**
Mobiluncus mulieris	0.00	0.00	0.00	0.32	**0.09**	0.00	0.00	0.00	0.06	**0.01**
Murdochiella asaccharolytica	0.03	0.00	0.00	0.00	**0.00**	0.00	0.00	0.00	0.00	**0.00**
Mycoplasma hominis	0.24	0.03	1.84	0.17	**0.49**	0.17	0.06	0.52	0.08	**0.20**
Neisseriaceae	0.00	0.00	0.06	0.00	**0.01**	0.03	0.00	0.02	0.00	**0.02**
Parabacteroides	0.00	0.00	0.00	0.00	**0.00**	0.00	0.00	0.01	0.00	**0.00**
Parabacteroides distasonis	0.00	0.00	0.00	0.00	**0.00**	0.00	0.01	0.00	0.00	**0.00**
Parvimonas micra	0.11	0.02	0.41	0.14	**0.15**	0.22	0.09	0.24	0.14	**0.16**
Peptoniphilus	0.30	0.00	0.03	0.00	**0.05**	0.44	0.05	0.01	0.15	**0.16**
Peptoniphilus asaccharolyticus	0.05	0.00	0.00	0.00	**0.01**	0.00	0.00	0.00	0.00	**0.00**
Peptoniphilus coxii	0.04	0.00	0.00	0.00	**0.01**	0.02	0.00	0.00	0.05	**0.02**

Peptoniphilus duerdenii	0.00	0.00	0.00	0.00	**0.00**	0.00	0.00	0.00	0.07	**0.02**
Peptoniphilus gorbachii	0.05	0.00	0.00	0.00	**0.01**	0.01	0.00	0.00	0.00	**0.00**
Peptoniphilus harei	0.00	0.00	0.00	0.00	**0.00**	0.10	0.00	0.00	0.13	**0.05**
Peptoniphilus indolicus	0.00	0.00	0.01	0.00	**0.00**	0.00	0.00	0.02	0.00	**0.00**
Peptoniphilus koenoeneniae	0.03	0.00	0.00	0.01	**0.01**	0.00	0.00	0.00	0.01	**0.00**
Peptoniphilus lacrimalis	0.07	0.00	0.04	0.02	**0.02**	0.02	0.00	0.02	0.02	**0.01**
Peptoniphilus tyrrelliae	0.00	0.00	0.00	0.00	**0.00**	0.04	0.00	0.00	0.00	**0.01**
Peptostreptococcus anaerobius	0.00	0.00	0.08	0.00	**0.02**	0.20	0.00	0.09	0.01	**0.05**
Peptostreptococcus russellii	0.00	0.00	0.00	0.00	**0.00**	0.00	0.00	0.00	0.00	**0.00**
Porphyromonas	0.00	0.00	0.03	0.00	**0.01**	0.00	0.00	0.01	0.07	**0.03**
Porphyromonas bennonis	0.21	0.00	0.00	0.00	**0.03**	0.14	0.02	0.00	0.03	**0.04**
Porphyromonas endodontalis	0.00	0.00	0.02	0.00	**0.00**	0.00	0.00	0.00	0.00	**0.00**
Porphyromonas somerae	0.00	0.00	0.00	0.00	**0.00**	0.00	0.00	0.00	0.06	**0.01**
Porphyromonas uenonis	0.21	0.03	0.22	0.03	**0.09**	0.05	0.03	0.11	0.08	**0.07**
Prevotella	0.01	0.11	0.01	0.34	**0.13**	0.09	0.20	0.03	0.25	**0.13**
Prevotella amnii	2.17	0.00	3.34	2.37	**1.96**	0.17	0.74	0.99	0.20	**0.58**
Prevotella bergensis	0.00	0.00	0.00	0.00	**0.00**	0.00	0.00	0.00	0.01	**0.00**
Prevotella bivia	2.30	0.47	2.84	0.79	**1.32**	0.91	0.52	1.10	1.12	**0.87**
Prevotella buccalis	0.22	0.00	0.76	1.79	**1.26**	0.08	0.41	0.66	1.77	**1.14**
Prevotella copri	0.00	0.00	0.00	0.00	**0.00**	0.00	0.01	0.01	0.00	**0.00**
Prevotella corporis	0.00	0.00	0.00	0.04	**0.01**	0.14	0.00	0.00	0.01	**0.03**
Prevotella disiens	0.03	0.06	0.05	0.02	**0.04**	1.08	0.04	0.05	0.18	**0.27**
Prevotella intermedia	0.00	0.00	0.00	0.00	**0.00**	0.01	0.00	0.00	0.00	**0.00**
Prevotella melaninogenica	0.06	0.00	2.23	0.02	**0.49**	0.00	0.03	0.51	0.01	**0.36**
Prevotella pallens	0.00	0.00	0.00	0.03	**0.01**	0.00	0.00	0.00	0.00	**0.00**
Prevotella salivae	0.00	0.00	0.00	0.00	**0.00**	0.07	0.00	0.00	0.14	**0.04**
Prevotella timonensis	1.56	0.30	5.04	3.10	**2.18**	0.96	0.37	3.21	0.31	**1.13**
Propionibacterium acnes	0.00	0.00	0.00	0.00	**0.00**	0.00	0.00	0.00	0.00	**0.00**
Propionimicrobium lymphophilum	0.00	0.00	0.00	0.00	**0.00**	0.00	0.00	0.00	0.00	**0.00**
Ralstonia insidiosa	0.02	0.00	0.00	0.00	**0.00**	0.00	0.00	0.00	0.00	**0.00**
Rikenellaceae	0.04	0.00	0.17	0.25	**0.12**	0.00	0.02	0.16	0.17	**0.10**
Roseburia	0.00	0.00	0.79	1.53	**0.69**	0.00	0.04	0.20	1.37	**0.47**
Ruminococcaceae	0.00	0.00	0.00	0.00	**0.00**	0.02	0.00	0.00	0.00	**0.00**
Ruminococcus albus	0.00	0.00	0.00	0.00	**0.00**	0.00	0.00	0.01	0.00	**0.00**
Ruminococcus flavefaciens	0.00	0.00	0.00	0.00	**0.00**	0.00	0.00	0.00	0.00	**0.00**
Ruminococcus torques	0.00	0.00	0.00	0.00	**0.00**	0.00	0.00	0.01	0.00	**0.00**
Saccharofermentans acetigenes	0.07	0.00	0.11	0.07	**0.05**	0.00	0.12	0.19	0.08	**0.11**
Saccharopolyspora	0.00	0.00	0.00	0.00	**0.00**	0.00	0.01	0.00	0.00	**0.00**
Senegalimassilia	0.18	0.10	0.40	0.73	**0.40**	0.06	0.22	0.67	0.32	**0.30**
Shuttleworthia	2.58	0.00	3.75	13.38	**5.56**	0.00	1.70	1.51	7.52	**3.17**
Sneathia	0.14	0.20	0.09	0.40	**0.20**	0.03	0.07	0.07	0.06	**0.08**
Sneathia sanguinegens	5.45	5.01	9.65	5.23	**5.98**	2.78	6.04	5.75	4.22	**5.04**
Solobacterium moorei	0.00	0.00	0.00	0.00	**0.00**	0.02	0.00	0.00	0.00	**0.00**
Staphylococcus aureus	7.14	0.01	0.00	0.00	**1.07**	0.00	0.00	0.00	0.00	**0.00**
Staphylococcus haemolyticus	0.00	0.00	0.00	0.00	**0.00**	0.01	0.00	0.00	0.00	**0.00**
Staphylococcus lugdunensis	0.00	0.00	0.00	0.00	**0.00**	0.00	0.00	0.00	0.00	**0.00**
Streptococcus	0.09	0.00	0.00	0.03	**0.02**	0.01	0.00	0.00	0.04	**0.01**
Streptococcus agalactiae	0.00	1.16	0.00	0.00	**0.25**	0.00	0.03	0.01	0.13	**1.07**
Streptococcus anginosus	0.11	0.00	0.06	0.00	**0.03**	2.17	0.39	0.09	0.03	**0.66**
Streptococcus constellatus	0.00	0.00	0.00	0.00	**0.00**	0.00	0.02	0.00	0.00	**0.00**
Streptococcus mitis	0.02	0.00	0.00	0.00	**0.00**	0.00	0.00	0.00	0.00	**0.00**
Streptococcus sanguinis	0.00	0.00	0.00	0.05	**0.01**	0.00	0.00	0.00	0.00	**0.00**
Sutterella stercoricanis	0.00	0.00	0.02	0.00	**0.00**	0.01	0.00	0.01	0.01	**0.01**
Sutterella wadsworthensis	0.00	0.00	0.00	0.00	**0.00**	0.00	0.00	0.00	0.01	**0.00**
Tepidimicrobium	0.00	0.00	0.00	0.00	**0.00**	0.00	0.00	0.00	0.00	**0.00**
Trueperella	0.00	0.00	0.05	0.01	**0.01**	0.00	0.00	0.06	0.00	**0.02**
Uncultured bacterium 17.002.2	0.00	0.00	0.00	0.00	**0.00**	0.00	0.00	0.00	0.00	**0.00**
Uncultured bacterium 24.053.1	0.00	0.00	0.00	0.01	**0.00**	0.00	0.00	0.00	0.00	**0.00**
Uncultured bacterium 505T 18133	0.00	0.00	0.08	0.00	**0.02**	0.00	0.00	0.05	0.00	**0.01**
Uncultured bacterium 5669	0.00	0.00	0.00	0.00	**0.00**	0.00	0.00	0.00	0.00	**0.00**

Uncultured bacterium 5669ncd431b01c1	0.05	0.00	0.00	0.00	**0.01**	0.00	0.00	0.00	0.00	**0.00**
Uncultured bacterium BD04564	0.00	0.00	0.00	0.00	**0.00**	0.00	0.00	0.00	0.00	**0.00**
Uncultured bacterium BFV02 159	0.00	0.00	0.00	0.00	**0.00**	0.00	0.00	0.00	0.06	**0.01**
Ureaplasma	0.01	0.03	0.00	0.00	**0.01**	0.05	0.16	0.03	0.01	**0.07**
Ureaplasma parvum	0.00	0.02	0.00	0.00	**0.01**	0.03	0.04	0.01	0.01	**0.02**
Ureaplasma urealyticum	0.01	0.00	0.15	0.01	**0.04**	0.06	0.00	0.18	0.00	**0.06**
Varibaculum cambriense	0.00	0.00	0.00	0.00	**0.00**	0.02	0.00	0.00	0.23	**0.06**
Veillonella montpellierensis	0.74	0.00	0.02	0.00	**0.13**	0.27	0.03	0.06	0.01	**0.07**
Veillonellaceae	0.03	0.02	0.00	0.03	**0.02**	0.00	0.04	0.02	0.12	**0.05**

Table S7. Taxa prevalence by urbanization level and ethnicity by body site. Cells are colored in a gradient from lower (blue) to higher (red) values.

	Prevalence of each taxa per group (%)					p value							p value	
	**Cervicovaginal site**							**Introitus**						
	Amerindian urbanization groups							Amerindian urbanization groups						
Taxa	Low	Medium	High	Mestizos	**Total**	Amerindian urban groups	Ethnicity	Low	Medium	High	Mestizos	**Total**	Amerindian urban groups	Ethnicity
N women	15	20	20	27	**95**			18	22	23	24	**101**		
Actinomyces turicensis	6.7	0.0	0.0	0.0	**1.1**	ns	ns	11.1	0.0	0.0	4.2	**4.0**	ns	ns
Aerococcus christensenii	6.7	0.0	15.0	3.7	**11.6**	ns	ns	0.0	9.1	18.2	20.8	**16.8**	ns	ns
Aggregatibacter aphrophilus	0.0	0.0	0.0	0.0	**0.0**	ns	ns	0.0	4.5	0.0	0.0	**1.0**	ns	ns
Alloprevotella rava	6.7	0.0	0.0	0.0	**1.1**	ns	ns	0.0	0.0	0.0	0.0	**1.0**	ns	ns
Alloscardovia omnicolens	0.0	0.0	0.0	0.0	**0.0**	ns	ns	0.0	4.5	0.0	0.0	**1.0**	ns	ns
Anaerococcus	0.0	0.0	0.0	0.0	**0.0**	ns	ns	11.1	0.0	0.0	0.0	**2.0**	ns	ns
Anaerococcus hydrogenalis	0.0	0.0	0.0	0.0	**0.0**	ns	ns	11.1	4.5	0.0	8.3	**5.9**	ns	ns
Anaerococcus lactolyticus	6.7	0.0	0.0	0.0	**1.1**	ns	ns	11.1	4.5	9.1	4.2	**6.9**	ns	ns
Anaerococcus murdochii	6.7	0.0	0.0	0.0	**1.1**	ns	ns	5.6	0.0	0.0	0.0	**2.0**	ns	ns
Anaerococcus obesiensis	13.3	0.0	0.0	0.0	**2.1**	ns	ns	5.6	0.0	0.0	4.2	**3.0**	ns	ns
Anaerococcus octavius	0.0	0.0	0.0	0.0	**0.0**	ns	ns	11.1	4.5	0.0	4.2	**4.0**	ns	ns
Anaerococcus prevotii	6.7	0.0	0.0	0.0	**2.1**	ns	ns	5.6	0.0	4.5	0.0	**4.0**	ns	ns
Anaerococcus tetradius	0.0	0.0	15.0	0.0	**3.2**	ns	ns	0.0	0.0	13.6	4.2	**5.0**	ns	ns
Anaerococcus vaginalis	0.0	0.0	0.0	0.0	**0.0**	ns	ns	0.0	4.5	0.0	0.0	**2.0**	ns	ns
Arcanobacterium	0.0	0.0	0.0	0.0	**0.0**	ns	ns	0.0	0.0	0.0	0.0	**1.0**	ns	ns
Arcanobacterium phocae	6.7	0.0	0.0	0.0	**1.1**	ns	ns	0.0	0.0	0.0	0.0	**0.0**	ns	ns
Arthrobacter albus	0.0	0.0	0.0	0.0	**0.0**	ns	ns	0.0	0.0	0.0	0.0	**1.0**	ns	ns
Arthrobacter cumminsii	0.0	0.0	0.0	0.0	**0.0**	ns	ns	0.0	0.0	0.0	0.0	**1.0**	ns	ns
Atopobium parvulum	0.0	0.0	5.0	0.0	**1.1**	ns	ns	0.0	0.0	0.0	0.0	**0.0**	ns	ns
Atopobium vaginae	40.0	35.0	55.0	55.6	**51.6**	ns	ns	44.4	54.5	50.0	62.5	**57.4**	ns	ns
Bacteroidales	0.0	5.0	5.0	3.7	**3.2**	ns	ns	0.0	4.5	4.5	8.3	**5.9**	ns	ns
Bacteroides coagulans	6.7	0.0	0.0	0.0	**1.1**	ns	ns	11.1	0.0	4.5	4.2	**5.0**	ns	ns
Bacteroides uniformis	0.0	0.0	0.0	0.0	**0.0**	ns	ns	0.0	0.0	4.5	0.0	**1.0**	ns	ns
Bifidobacterium bifidum	0.0	0.0	0.0	0.0	**0.0**	ns	ns	0.0	4.5	4.5	4.2	**3.0**	ns	ns
Bifidobacterium breve	0.0	0.0	5.0	0.0	**1.1**	ns	ns	0.0	0.0	4.5	0.0	**1.0**	ns	ns
Bifidobacterium longum	0.0	0.0	5.0	3.7	**2.1**	ns	ns	0.0	4.5	4.5	4.2	**3.0**	ns	ns
Brachybacterium alimentarium	6.7	5.0	10.0	0.0	**4.2**	ns	ns	16.7	4.5	4.5	4.2	**7.9**	ns	ns
Brachybacterium tyrofermentans	13.3	10.0	10.0	0.0	**6.3**	ns	ns	0.0	9.1	4.5	8.3	**5.9**	ns	ns
Brevibacterium	6.7	5.0	0.0	0.0	**2.1**	ns	ns	0.0	4.5	4.5	0.0	**2.0**	ns	ns
Brevibacterium linens	13.3	35.0	45.0	3.7	**23.2**	ns	*	33.3	59.1	36.4	16.7	**37.6**	ns	ns
Brevibacterium massiliense	6.7	0.0	0.0	0.0	**1.1**	ns	ns	0.0	0.0	0.0	0.0	**2.0**	ns	ns
Bulleidia extructa	6.7	0.0	5.0	3.7	**3.2**	ns	ns	0.0	4.5	4.5	0.0	**3.0**	ns	ns
Campylobacter	0.0	0.0	0.0	0.0	**0.0**	ns	ns	0.0	0.0	0.0	4.2	**1.0**	ns	ns
Campylobacter faecalis	0.0	0.0	5.0	0.0	**1.1**	ns	ns	0.0	4.5	4.5	4.2	**3.0**	ns	ns
Campylobacter ureolyticus	13.3	0.0	5.0	3.7	**4.2**	ns	ns	11.1	0.0	13.6	4.2	**6.9**	ns	ns
Clostridiales	13.3	5.0	5.0	3.7	**6.3**	ns	ns	5.6	13.6	13.6	8.3	**12.9**	ns	ns
Clostridium	6.7	25.0	25.0	37.0	**27.4**	ns	ns	5.6	27.3	31.8	25.0	**22.8**	ns	ns
Clostridium perfringens	0.0	0.0	0.0	3.7	**1.1**	ns	ns	0.0	0.0	0.0	0.0	**0.0**	ns	ns
Clostridium stercorarium	0.0	5.0	0.0	0.0	**1.1**	ns	ns	0.0	4.5	4.5	4.2	**4.0**	ns	ns
Comamonadaceae	0.0	0.0	0.0	0.0	**0.0**	ns	ns	0.0	0.0	4.5	0.0	**2.0**	ns	ns
Coriobacteriaceae	0.0	0.0	5.0	0.0	**2.1**	ns	ns	0.0	4.5	4.5	4.2	**3.0**	ns	ns
Corynebacteriaceae	0.0	0.0	0.0	0.0	**0.0**	ns	ns	5.6	4.5	0.0	4.2	**3.0**	ns	ns
Corynebacterium	6.7	0.0	0.0	0.0	**1.1**	ns	ns	16.7	0.0	13.6	4.2	**10.9**	ns	ns
Corynebacterium afermentans	0.0	0.0	0.0	0.0	**0.0**	ns	ns	0.0	0.0	0.0	0.0	**1.0**	ns	ns
Corynebacterium amycolatum	6.7	0.0	0.0	0.0	**1.1**	ns	ns	22.2	13.6	4.5	8.3	**11.9**	ns	ns

Corynebacterium atypicum	0.0	0.0	0.0	0.0	**0.0**	ns	ns	0.0	4.5	0.0	0.0	**1.0**	ns	ns
Corynebacterium aurimucosum	6.7	0.0	0.0	0.0	**1.1**	ns	ns	5.6	4.5	4.5	8.3	**6.9**	ns	ns
Corynebacterium coyleae	0.0	0.0	0.0	0.0	**0.0**	ns	ns	0.0	0.0	0.0	0.0	**1.0**	ns	ns
Corynebacterium freneyi	0.0	5.0	0.0	0.0	**1.1**	ns	ns	0.0	4.5	0.0	0.0	**1.0**	ns	ns
Corynebacterium glucuronolyticum	0.0	0.0	0.0	0.0	**1.1**	ns	ns	0.0	0.0	0.0	0.0	**0.0**	ns	ns
Corynebacterium jeikeium	6.7	0.0	0.0	0.0	**1.1**	ns	ns	5.6	0.0	0.0	0.0	**4.0**	ns	ns
pseudogenitalium	0.0	0.0	0.0	0.0	**0.0**	ns	ns	11.1	0.0	0.0	4.2	**3.0**	ns	ns
pyruviciproducens	6.7	0.0	0.0	0.0	**1.1**	ns	ns	5.6	4.5	0.0	0.0	**3.0**	ns	ns
Corynebacterium riegelii	0.0	0.0	0.0	0.0	**0.0**	ns	ns	0.0	0.0	0.0	0.0	**1.0**	ns	ns
Corynebacterium simulans	0.0	0.0	0.0	0.0	**0.0**	ns	ns	0.0	9.1	0.0	8.3	**4.0**	ns	ns
Corynebacterium striatum	0.0	0.0	0.0	0.0	**0.0**	ns	ns	0.0	9.1	0.0	8.3	**5.0**	ns	ns
Corynebacterium sundsvallense	0.0	0.0	0.0	0.0	**0.0**	ns	ns	0.0	0.0	0.0	0.0	**2.0**	ns	ns
Corynebacterium tuscaniense	0.0	0.0	0.0	0.0	**0.0**	ns	ns	0.0	0.0	0.0	0.0	**1.0**	ns	ns
Corynebacterium urealyticum	0.0	0.0	0.0	0.0	**0.0**	ns	ns	0.0	0.0	0.0	0.0	**1.0**	ns	ns
Cystobacter	0.0	0.0	0.0	0.0	**0.0**	ns	ns	0.0	0.0	0.0	0.0	**1.0**	ns	ns
Dermabacter hominis	0.0	0.0	0.0	0.0	**0.0**	ns	ns	0.0	0.0	0.0	0.0	**1.0**	ns	ns
Dialister	0.0	0.0	5.0	7.4	**3.2**	ns	ns	5.6	0.0	0.0	4.2	**2.0**	ns	ns
Dialister micraerophilus	0.0	0.0	25.0	33.3	**16.8**	*	ns	22.2	13.6	27.3	29.2	**25.7**	ns	ns
Dialister propionicifaciens	6.7	0.0	0.0	0.0	**1.1**	ns	ns	0.0	0.0	0.0	4.2	**1.0**	ns	ns
Dietzia	6.7	10.0	15.0	0.0	**6.3**	ns	ns	16.7	13.6	13.6	8.3	**12.9**	ns	ns
Dorea longicatena	0.0	0.0	0.0	0.0	**0.0**	ns	ns	0.0	0.0	4.5	0.0	**1.0**	ns	ns
Escherichia.Shigella	0.0	0.0	0.0	0.0	**0.0**	ns	ns	0.0	4.5	0.0	0.0	**1.0**	ns	ns
Eubacterium oxidoreducens	0.0	0.0	0.0	0.0	**0.0**	ns	ns	0.0	0.0	4.5	0.0	**1.0**	ns	ns
Eubacterium rectale	0.0	0.0	0.0	0.0	**0.0**	ns	ns	0.0	0.0	4.5	0.0	**1.0**	ns	ns
Eubacterium yurii	6.7	0.0	5.0	0.0	**2.1**	ns	ns	0.0	0.0	0.0	4.2	**1.0**	ns	ns
Ezakiella	6.7	0.0	0.0	0.0	**1.1**	ns	ns	0.0	0.0	0.0	0.0	**0.0**	ns	ns
Facklamia hominis	0.0	0.0	0.0	0.0	**0.0**	ns	ns	5.6	0.0	0.0	0.0	**1.0**	ns	ns
Faecalibacterium prausnitzii	0.0	0.0	0.0	0.0	**0.0**	ns	ns	0.0	4.5	4.5	0.0	**2.0**	ns	ns
Fastidiosipila	0.0	5.0	10.0	3.7	**5.3**	ns	ns	0.0	13.6	9.1	4.2	**5.9**	ns	ns
Finegoldia magna	6.7	0.0	0.0	0.0	**2.1**	ns	ns	22.2	13.6	4.5	12.5	**12.9**	ns	ns
Fusobacterium	0.0	0.0	5.0	0.0	**1.1**	ns	ns	0.0	0.0	0.0	0.0	**0.0**	ns	ns
Fusobacterium gonidiaformans	0.0	0.0	5.0	0.0	**1.1**	ns	ns	0.0	0.0	0.0	0.0	**0.0**	ns	ns
Fusobacterium naviforme	0.0	0.0	5.0	3.7	**2.1**	ns	ns	5.6	0.0	4.5	4.2	**3.0**	ns	ns
Fusobacterium nucleatum	0.0	0.0	0.0	3.7	**1.1**	ns	ns	0.0	0.0	0.0	8.3	**2.0**	ns	ns
Gardnerella vaginalis	53.3	75.0	80.0	81.5	**80.0**	ns	ns	72.2	90.9	72.7	83.3	**86.1**	ns	ns
Gemella asaccharolytica	0.0	5.0	5.0	0.0	**4.2**	ns	ns	5.6	13.6	13.6	4.2	**9.9**	ns	ns
Gemella haemolysans	13.3	0.0	0.0	3.7	**3.2**	ns	ns	0.0	0.0	0.0	0.0	**0.0**	ns	ns
Gordonibacter pamelaeae	0.0	0.0	0.0	0.0	**0.0**	ns	ns	11.1	0.0	0.0	0.0	**2.0**	ns	ns
Granulicatella elegans	13.3	0.0	5.0	0.0	**3.2**	ns	ns	11.1	0.0	9.1	0.0	**5.0**	ns	ns
Haemophilus haemolyticus	6.7	0.0	0.0	0.0	**1.1**	ns	ns	5.6	0.0	0.0	0.0	**1.0**	ns	ns
Haemophilus parainfluenzae	0.0	0.0	0.0	0.0	**0.0**	ns	ns	0.0	0.0	0.0	4.2	**1.0**	ns	ns
Helcococcus sueciensis	0.0	0.0	0.0	0.0	**0.0**	ns	ns	0.0	0.0	0.0	0.0	**1.0**	ns	ns
Howardella ureilytica	0.0	0.0	0.0	3.7	**1.1**	ns	ns	0.0	0.0	0.0	4.2	**1.0**	ns	ns
Lachnospira	6.7	0.0	5.0	3.7	**4.2**	ns	ns	0.0	4.5	0.0	4.2	**2.0**	ns	ns
Lachnospiraceae	0.0	0.0	0.0	0.0	**0.0**	ns	ns	0.0	4.5	0.0	0.0	**1.0**	ns	ns
Lactobacillus crispatus	13.3	25.0	5.0	14.8	**14.7**	ns	ns	11.1	31.8	4.5	16.7	**15.8**	ns	ns
Lactobacillus equicursoris	0.0	5.0	0.0	0.0	**1.1**	ns	ns	0.0	0.0	0.0	0.0	**0.0**	ns	ns
Lactobacillus gasseri	0.0	5.0	0.0	0.0	**1.1**	ns	ns	0.0	4.5	4.5	0.0	**2.0**	ns	ns
Lactobacillus iners	73.3	45.0	40.0	74.1	**64.2**	*	ns	72.2	72.7	68.2	83.3	**73.3**	ns	ns
Lactobacillus jensenii	6.7	10.0	0.0	7.4	**5.3**	ns	ns	0.0	9.1	0.0	12.5	**5.0**	ns	ns
Lactobacillus vaginalis	0.0	0.0	0.0	0.0	**0.0**	ns	ns	0.0	0.0	0.0	4.2	**1.0**	ns	ns
Leptotrichia amnionii	13.3	20.0	25.0	33.3	**27.4**	ns	ns	16.7	22.7	22.7	29.2	**23.8**	ns	ns
Leucobacter komagatae	0.0	5.0	0.0	0.0	**1.1**	ns	ns	0.0	0.0	0.0	0.0	**0.0**	ns	ns
Megasphaera	6.7	5.0	20.0	25.9	**16.8**	ns	ns	5.6	4.5	18.2	25.0	**15.8**	ns	ns
Megasphaera micronuciformis	20.0	20.0	40.0	51.9	**37.9**	ns	ns	11.1	27.3	40.9	50.0	**36.6**	ns	ns
Megasphaera sueciensis	0.0	0.0	0.0	0.0	**0.0**	ns	ns	0.0	0.0	0.0	0.0	**1.0**	ns	ns

Mobiluncus curtisii	6.7	0.0	5.0	3.7	**3.2**	ns	ns	0.0	0.0	4.5	0.0	**1.0**	ns	ns
Mobiluncus mulieris	0.0	0.0	0.0	25.9	**7.4**	ns	*	0.0	0.0	0.0	16.7	**4.0**	ns	ns
Murdochiella asaccharolytica	6.7	0.0	0.0	0.0	**1.1**	ns	ns	0.0	0.0	0.0	0.0	**1.0**	ns	ns
Mycoplasma hominis	20.0	5.0	20.0	37.0	**22.1**	ns	ns	5.6	13.6	36.4	33.3	**25.7**	ns	ns
Neisseriaceae	0.0	0.0	5.0	0.0	**1.1**	ns	ns	11.1	0.0	9.1	0.0	**5.0**	ns	ns
Parabacteroides	0.0	0.0	0.0	0.0	**0.0**	ns	ns	0.0	0.0	4.5	0.0	**1.0**	ns	ns
Parabacteroides distasonis	0.0	0.0	0.0	0.0	**0.0**	ns	ns	0.0	4.5	4.5	0.0	**2.0**	ns	ns
Parvimonas micra	26.7	5.0	40.0	40.7	**27.4**	*	ns	22.2	18.2	36.4	33.3	**27.7**	ns	ns
Peptoniphilus	6.7	0.0	5.0	0.0	**2.1**	ns	ns	16.7	9.1	9.1	12.5	**10.9**	ns	ns
Peptoniphilus asaccharolyticus	6.7	0.0	0.0	0.0	**1.1**	ns	ns	0.0	0.0	0.0	0.0	**0.0**	ns	ns
Peptoniphilus coxii	6.7	0.0	0.0	0.0	**1.1**	ns	ns	5.6	0.0	0.0	8.3	**4.0**	ns	ns
Peptoniphilus duerdenii	0.0	0.0	0.0	0.0	**0.0**	ns	ns	0.0	0.0	0.0	4.2	**1.0**	ns	ns
Peptoniphilus gorbachii	6.7	0.0	0.0	0.0	**1.1**	ns	ns	5.6	0.0	0.0	0.0	**1.0**	ns	ns
Peptoniphilus harei	0.0	0.0	0.0	0.0	**0.0**	ns	ns	5.6	0.0	0.0	8.3	**3.0**	ns	ns
Peptoniphilus indolicus	0.0	0.0	10.0	7.4	**4.2**	ns	ns	0.0	0.0	9.1	4.2	**3.0**	ns	ns
Peptoniphilus koenoeneniae	13.3	0.0	0.0	7.4	**4.2**	ns	ns	5.6	0.0	4.5	8.3	**4.0**	ns	ns
Peptoniphilus lacrimalis	6.7	0.0	15.0	3.7	**6.3**	ns	*	5.6	0.0	9.1	12.5	**6.9**	ns	ns
Peptoniphilus tyrrelliae	0.0	0.0	0.0	0.0	**0.0**	ns	ns	5.6	0.0	0.0	0.0	**1.0**	ns	ns
Peptostreptococcus anaerobius	6.7	0.0	5.0	0.0	**2.1**	ns	ns	16.7	0.0	13.6	4.2	**6.9**	ns	ns
Peptostreptococcus russellii	0.0	0.0	0.0	0.0	**0.0**	ns	ns	0.0	0.0	0.0	4.2	**1.0**	ns	ns
Porphyromonas	0.0	0.0	5.0	3.7	**2.1**	ns	ns	0.0	0.0	4.5	4.2	**3.0**	ns	ns
Porphyromonas bennonis	6.7	0.0	0.0	0.0	**1.1**	ns	ns	11.1	4.5	4.5	4.2	**5.9**	ns	ns
Porphyromonas endodontalis	0.0	0.0	5.0	0.0	**1.1**	ns	ns	0.0	0.0	0.0	0.0	**0.0**	ns	ns
Porphyromonas somerae	0.0	0.0	0.0	0.0	**0.0**	ns	ns	0.0	0.0	0.0	4.2	**1.0**	ns	ns
Porphyromonas uenonis	20.0	5.0	15.0	14.8	**11.6**	ns	ns	5.6	4.5	18.2	12.5	**11.9**	ns	ns
Prevotella	6.7	5.0	5.0	29.6	**12.6**	ns	ns	11.1	9.1	4.5	33.3	**17.8**	ns	ns
Prevotella amnii	13.3	5.0	20.0	33.3	**21.1**	ns	ns	11.1	9.1	13.6	12.5	**15.8**	ns	ns
Prevotella bergensis	0.0	0.0	0.0	0.0	**0.0**	ns	ns	0.0	0.0	0.0	4.2	**1.0**	ns	ns
Prevotella bivia	26.7	5.0	30.0	18.5	**21.1**	ns	ns	27.8	22.7	36.4	20.8	**28.7**	ns	ns
Prevotella buccalis	13.3	0.0	20.0	37.0	**21.1**	ns	ns	5.6	4.5	18.2	41.7	**21.8**	ns	ns
Prevotella copri	0.0	0.0	0.0	0.0	**0.0**	ns	ns	0.0	4.5	4.5	0.0	**2.0**	ns	ns
Prevotella corporis	0.0	0.0	0.0	11.1	**4.2**	ns	ns	5.6	0.0	0.0	4.2	**2.0**	ns	ns
Prevotella disiens	6.7	5.0	10.0	3.7	**6.3**	ns	ns	11.1	4.5	13.6	12.5	**10.9**	ns	ns
Prevotella intermedia	0.0	0.0	0.0	0.0	**0.0**	ns	ns	5.6	0.0	0.0	0.0	**1.0**	ns	ns
Prevotella melaninogenica	6.7	0.0	15.0	3.7	**7.4**	ns	ns	0.0	9.1	4.5	4.2	**5.9**	ns	ns
Prevotella pallens	0.0	0.0	0.0	3.7	**1.1**	ns	ns	0.0	0.0	0.0	0.0	**0.0**	ns	ns
Prevotella salivae	0.0	0.0	0.0	0.0	**0.0**	ns	ns	5.6	0.0	0.0	8.3	**3.0**	ns	ns
Prevotella timonensis	13.3	20.0	30.0	44.4	**27.4**	ns	ns	11.1	22.7	36.4	29.2	**23.8**	ns	ns
Propionibacterium acnes	0.0	0.0	0.0	0.0	**0.0**	ns	ns	0.0	4.5	4.5	0.0	**2.0**	ns	ns
Propionimicrobium lymphophilum	0.0	0.0	0.0	0.0	**0.0**	ns	ns	0.0	0.0	0.0	0.0	**1.0**	ns	ns
Ralstonia insidiosa	6.7	0.0	0.0	0.0	**1.1**	ns	ns	0.0	0.0	0.0	0.0	**0.0**	ns	ns
Rikenellaceae	6.7	0.0	10.0	22.2	**10.5**	ns	ns	0.0	4.5	9.1	20.8	**10.9**	ns	ns
Roseburia	0.0	0.0	15.0	7.4	**7.4**	ns	ns	0.0	4.5	4.5	4.2	**4.0**	ns	ns
Ruminococcaceae	0.0	0.0	0.0	0.0	**0.0**	ns	ns	5.6	0.0	4.5	0.0	**2.0**	ns	ns
Ruminococcus albus	0.0	0.0	0.0	0.0	**0.0**	ns	ns	0.0	0.0	4.5	0.0	**1.0**	ns	ns
Ruminococcus flavefaciens	0.0	0.0	0.0	0.0	**0.0**	ns	ns	0.0	4.5	0.0	0.0	**1.0**	ns	ns
Ruminococcus torques	0.0	0.0	0.0	0.0	**0.0**	ns	ns	0.0	0.0	4.5	0.0	**1.0**	ns	ns
Saccharofermentans acetigenes	6.7	0.0	20.0	11.1	**8.4**	ns	ns	0.0	4.5	18.2	12.5	**10.9**	ns	ns
Saccharopolyspora	0.0	0.0	0.0	0.0	**0.0**	ns	ns	0.0	4.5	0.0	0.0	**1.0**	ns	ns
Senegalimassilia	20.0	15.0	30.0	55.6	**35.8**	ns	ns	11.1	22.7	36.4	45.8	**32.7**	ns	ns
Shuttleworthia	20.0	0.0	20.0	44.4	**23.2**	ns	ns	0.0	13.6	13.6	37.5	**22.8**	ns	ns
Sneathia	13.3	10.0	15.0	25.9	**16.8**	ns	ns	5.6	13.6	18.2	12.5	**12.9**	ns	ns
Sneathia sanguinegens	40.0	15.0	60.0	59.3	**48.4**	*	ns	27.8	27.3	45.5	58.3	**45.5**	ns	ns
Solobacterium moorei	0.0	0.0	0.0	0.0	**0.0**	ns	ns	5.6	0.0	0.0	0.0	**1.0**	ns	ns
Staphylococcus aureus	6.7	5.0	0.0	0.0	**3.2**	ns	ns	0.0	0.0	0.0	0.0	**0.0**	ns	ns
Staphylococcus haemolyticus	0.0	0.0	0.0	0.0	**0.0**	ns	ns	5.6	0.0	0.0	0.0	**2.0**	ns	ns

Staphylococcus lugdunensis	0.0	0.0	0.0	0.0	**0.0**	ns	ns	0.0	0.0	0.0	0.0	**1.0**	ns	ns
Streptococcus	6.7	0.0	0.0	3.7	**2.1**	ns	ns	5.6	0.0	0.0	4.2	**2.0**	ns	ns
Streptococcus agalactiae	0.0	15.0	0.0	0.0	**4.2**	ns	ns	0.0	9.1	4.5	4.2	**5.9**	ns	ns
Streptococcus anginosus	13.3	0.0	10.0	0.0	**4.2**	ns	ns	33.3	18.2	13.6	4.2	**16.8**	ns	ns
Streptococcus constellatus	0.0	0.0	0.0	0.0	**0.0**	ns	ns	0.0	4.5	0.0	0.0	**1.0**	ns	ns
Streptococcus mitis	6.7	0.0	0.0	0.0	**1.1**	ns	ns	0.0	0.0	0.0	0.0	**0.0**	ns	ns
Streptococcus sanguinis	0.0	0.0	0.0	3.7	**1.1**	ns	ns	0.0	0.0	0.0	0.0	**0.0**	ns	ns
Sutterella stercoricanis	0.0	0.0	10.0	0.0	**2.1**	ns	ns	11.1	0.0	4.5	4.2	**4.0**	ns	ns
Sutterella wadsworthensis	0.0	0.0	0.0	0.0	**0.0**	ns	ns	0.0	0.0	0.0	4.2	**1.0**	ns	ns
Tepidimicrobium	0.0	0.0	0.0	0.0	**0.0**	ns	ns	0.0	0.0	0.0	4.2	**1.0**	ns	ns
Trueperella	6.7	0.0	10.0	3.7	**4.2**	ns	ns	0.0	0.0	4.5	0.0	**2.0**	ns	ns
Uncultured bacterium 17.002.2	0.0	0.0	0.0	0.0	**1.1**	ns	ns	0.0	0.0	0.0	0.0	**0.0**	ns	ns
Uncultured bacterium 24.053.1	0.0	0.0	0.0	3.7	**1.1**	ns	ns	0.0	0.0	0.0	0.0	**0.0**	ns	ns
Uncultured bacterium 505T 18133	0.0	0.0	5.0	0.0	**1.1**	ns	ns	0.0	0.0	4.5	0.0	**1.0**	ns	ns
Uncultured bacterium 5669	0.0	0.0	0.0	0.0	**0.0**	ns	ns	0.0	0.0	0.0	4.2	**1.0**	ns	ns
Uncultured bacterium 5669ncd431b01c1	6.7	0.0	0.0	0.0	**1.1**	ns	ns	0.0	0.0	0.0	0.0	**0.0**	ns	ns
Uncultured bacterium BD04564	0.0	0.0	0.0	0.0	**0.0**	ns	ns	0.0	0.0	4.5	0.0	**1.0**	ns	ns
Uncultured bacterium BFV02 159	0.0	0.0	0.0	0.0	**0.0**	ns	ns	5.6	0.0	0.0	4.2	**2.0**	ns	ns
Ureaplasma	6.7	10.0	0.0	3.7	**6.3**	ns	ns	22.2	22.7	9.1	12.5	**16.8**	ns	ns
Ureaplasma parvum	0.0	10.0	0.0	3.7	**5.3**	ns	ns	5.6	18.2	9.1	16.7	**13.9**	ns	ns
Ureaplasma urealyticum	6.7	0.0	15.0	7.4	**8.4**	*	ns	11.1	0.0	13.6	4.2	**6.9**	ns	ns
Varibaculum cambriense	0.0	0.0	0.0	0.0	**0.0**	ns	ns	5.6	0.0	0.0	8.3	**3.0**	ns	ns
Veillonella montpellierensis	6.7	0.0	10.0	0.0	**5.3**	ns	ns	5.6	9.1	9.1	4.2	**6.9**	ns	ns
Veillonellaceae	6.7	5.0	0.0	3.7	**4.2**	ns	ns	0.0	9.1	4.5	16.7	**7.9**	ns	ns

& Fisher's exact test among Low, Medium, High and Mestizo groups. p-values <0.05 has are shown with *. ns indicate not significant differences

Table S8: Cervicovaginal shared and unique taxa among woman groups.

Comparison	Woman groups	total	taxon names
Among Amerindian urbanization groups	Low, Medium and High	30	*Porphyromonas_uenonis, Parvimonas_micra, Megasphaera_micronuciformis, Lactobacillus_jensenii, Ureaplasma_parvum, Mycoplasma_hominis, Prevotella_disiens, Lactobacillus_iners, Corynebacterium, Prevotella_bivia, Megasphaera, Senegalimassilia, Sneathia, Prevotella, Lactobacillus_amnionii, Clostridiales, Clostridium, Veillonellaceae, Gardnerella_vaginalis, Atopobium_vaginae, Brevibacterium_linens, Shuttleworthia, Dietzia, Prevotella_timonensis, Brachybacterium_alimentarium, Brachybacterium_tyrofermentans, Sneathia_sanguinegens, Prevotella_buccalis, Lactobacillus_crispatus, Prevotella_amnii*
	Low, High	15	*Streptococcus_anginosus, Granulicatella_elegans, Rikenellaceae, Campylobacter_ureolyticus, Saccharofermentans_acetigenes, Peptoniphilus, Prevotella_melaninogenica, Veillonella_montpellierensis, Trueperella, Peptostreptococcus_anaerobius, Peptoniphilus_lacrimalis, Lachnospira, Aerococcus_christensenii, Mobiluncus_curtisii, Eubacterium_yurii*
	Medium, High	12	*Gemella_asaccharolytica, Fastidiosipila, Roseburia, Corynebacterium_freneyi, Streptococcus_agalactiae, Clostridium_stercorarium, Dialister_micraerophilus, Ureaplasma, Lactobacillus_gasseri, Lactobacillus_equicursoris, Bacteroidales, Leucobacter_komagatae*
	Low	32	*Streptococcus, Anaerococcus_lactolyticus, Bulleidia_extructa, Prevotella_bergensis, Dialister_propionicifaciens, Ezakiella, Peptoniphilus_gorbachii, Anaerococcus_murdochii, Brevibacterium_massiliense, Corynebacterium_amycolatum, Haemophilus_haemolyticus, Anaerococcus_obesiensis, Anaerococcus_octavius, Gemella_haemolysans, Arcanobacterium_phocae, Finegoldia_magna, Ralstonia_insidiosa, Corynebacterium_pyruviciproducens, Murdochiella, Streptococcus_sanguinis, Alloprevotella, Bacteroides, Corynebacterium_jeikeium, Porphyromonas_bennonis, Uncultured_bacterium_5669ncd431b01c1, Staphylococcus_aureus, Actinomyces_turicensis, Peptoniphilus_coxii, Corynebacterium_aurimucosum, Brevibacterium, Peptoniphilus_asaccharolyticus, Peptoniphilus_koenoeneniae*
	Medium	0	NA

	High	16	*Uncultured_bacterium_505T_18133, Bifidobacterium_longum, Peptoniphilus_indolicus, Fusobacterium_naviforme, Anaerococcus_tetradius, Bifidobacterium_bifidum, Campylobacter_concisus, Coriobacteriaceae, Ureaplasma_urealyticum, Sutterella_stercoricanis, Neisseriaceae, Dialister, Fusobacterium_gonidiaformans, Fusobacterium, Porphyromonas, Porphyromonas_endodontalis*
Ethnicity	Amerindians and mestizo	42	*Porphyromonas_uenonis, Parvimonas_micra, Megasphaera_micronuciformis, Lactobacillus_jensenii, Bifidobacterium_longum, Peptoniphilus_indolicus, Mycoplasma_hominis, Fastidiosipila, Fusobacterium_naviforme, Prevotella_disiens, Rikenellaceae, Lactobacillus_iners, Roseburia, Campylobacter_ureolyticus, Prevotella_bivia, Megasphaera, Senegalimassilia, Saccharofermentans_acetigenes, Dialister_micraerophilus, Sneathia, Prevotella, Clostridiales, Campylobacter_concisus, Prevotella_melaninogenica, Clostridium, Ureaplasma_urealyticum, Veillonella_montpellierensis, Gardnerella_vaginalis, Atopobium_vaginae, Dialister, Brevibacterium_linens, Shuttleworthia, Peptoniphilus_lacrimalis, Prevotella_timonensis, Lachnospira, Aerococcus_christensenii, Porphyromonas, Bacteroidales, Sneathia_sanguinegens, Prevotella_buccalis, Lactobacillus_crispatus, Prevotella_amnii*
	Amerindians	19	*Streptococcus_anginosus, Uncultured_bacterium_505T_18133, Gemella_asaccharolytica, Granulicatella_elegans, Streptococcus_agalactiae, Anaerococcus_tetradius, Peptoniphilus, Bifidobacterium_bifidum, Sutterella_stercoricanis, Trueperella, Peptostreptococcus_anaerobius, Neisseriaceae, Dietzia, Fusobacterium_gonidiaformans, Brachybacterium_alimentarium, Brachybacterium_tyrofermentans, Mobiluncus_curtisii, Eubacterium_yurii, Porphyromonas_endodontalis*
	Mestizos	16	*Streptococcus, Bulleidia_extructa, Ureaplasma_parvum, Prevotella_corporis, Fusobacterium_nucleatum, Gemella_haemolysans, Lactobacillus_amnionii, Howardella_ureilytica, Ureaplasma, Mobiluncus_mulieris, Clostridium_perfringens, Streptococcus_mitis, Veillonellaceae, Uncultured_bacterium_24_053_1, Prevotella_pallens, Peptoniphilus_koenoeneniae*

Table S9. Variables associated with cervicovaginal community state types (CSTs). Red values indicate p<0.05 or p.adj<0.05.

Variables	Prevalence % (n/N) Cervicovaginal CSTs CST-L.iners	CST-G.vaginalis	CST-div1	CST-div2	*p-value (p.adj*)*
**Crop gardening**	66.7 (22/33)	77.8 (14/18)	7.7 (1/13)	38.5 (5/13)	*$2x10^{-4}$ (0.004)*
**Smear count of ≥20% clue cells**	10.0 (4/40)	8.0 (2/25)	53.8 (7/13)	26.7 (4/15)	*0.003 (0.037)*
**Number sexual couple history**					*0.008 (0.079)*
More than one	80.5 (33/41)	46.2 (12/26)	46.2 (6/13)	80.0 (12/15)	
One	19.5 (8/41)	53.8 (14/26)	53.8 (7/13)	20.0 (3/15)	
**Vaginal pH range**					*0.014 (0.115)*
4-4.7	71.1 (27/38)	52.0 (13/25)	38.5 (5/13)	33.3 (4/12)	
4.8-5.4	13.2 (5/38)	28.0 (7/25)	61.5 (8/13)	41.7 (5/12)	
5.5-7	15.8 (6/38)	20.0 (5/25)	0.0 (0/13)	25.0 (3/12)	
**Breast feeding currently**	47.5 (19/40)	80.0 (20/25)	66.7 (8/12)	35.7 (5/14)	*0.017 (0.115)*
**History of sexual contact with Mestizo**	48.8 (20/41)	34.6 (9/26)	76.9 (10/13)	26.7 (4/15)	*0.035 (0.200)*
**Number sexual partner last 60 days**					*0.040 (0.200)*
None	7.5 (3/40)	26.9 (7/26)	7.7 (1/13)	33.3 (5/15)	
One	92.5 (37/40)	73.1 (19/26)	92.3 (12/13)	66.7 (10/15)	
**Ethnicity**					*0.049 (0.200)*
Mestizo	26.8 (11/41)	19.2 (5/26)	61.5 (8/13)	20.0 (3/15)	
Amerindians	73.2 (30/41)	80.8 (21/26)	38.5 (5/13)	80.0 (12/15)	

*Fisher´s exact test. Adjusted p-values for multiple comparisons were performed using false discovery rate Benjamini-Hochberg (fdr-BH) method.

Table S10. Variables significantly associated with introital community state types (CSTs). Red values indicate p<0.05 or p.adj<0.05.

Variables		Prevalence % (n/N)		*p-value (p.adj*)*
		**CST-L.iners**	**CST-div**	
**Vaginal pH range**				*$1x10^{-4}$ (0.001)*
	4.0-4.7	75.8 (25/33)	24.3 (9/37)	
	4.8-5.4	12.1 (4/33)	54.1 (20/37)	
	5.5-7.0	12.1 (4/33)	21.6 (8/37)	
**Cytological smear count of ≥20% clue cells**		5.3 (2/38)	27.5 (11/40)	*0.013 (0.162)*
**Number sexual partners last 60 days**				*0.011 (0.162)*
	One	91.9 (34/37)	67.5 (27/40)	
	None	8.1 (3/37)	32.5 (13/40)	

*Adjusted p-values for multiple comparisons were performed using false discovery rate Benjamini-Hochberg (fdr-BH) method.

Table S11. Prevalence of dominant *Lactobacillus* sp. (>50% of relative abundance) in cervicovaginal microbiota by urbanization level and ethnicity.

	**Prevalence % (n)**				
	**Amerindians**				
	**Urbanization groups (N=55)**			**Amer-indians**	
**Profiles**	**Low (n=15)**	**Medium (n=20)**	**High (n=20)**	**(N=68)**	**Mestizos (N=27)**
**Diverse**	40.0 (6)	40.9 (8)	70.0 (14)	51.5 (35)	59.3 (16)
***L. iners* ***	60.0 (9)	45.0 (9)	25.0 (5)	42.6 (29)	40.7 (11)
***L. crispatus* ***	0.0 (0)	10.0 (2)	5.0 (1)	4.4 (3)	0.0 (0)
***L. gasseri* ***	0.0 (0)	5.0 (1)	0.0 (0)	1.5 (1)	0.0 (0)
Any *Lactobacillus* sp. dominance	60.0 (9)	60.0 (12)	30.0 (6)	48.5 (33)	40.7 (11)

NA cells indicate that statistical test could not be performed due to small n per

* >50% of relative abundance

Table S12. Cervicovaginal microbiota beta and alpha diversity metrics among all women by HPV status

	p value*			
**Group comparison**	**Bray distance to group centroid**	**R^2**	**Shannon**	**Simpson**
**HPV status** (Pos vs Neg**)**	0.495	0.009	0.496	0.588
**High-risk HPV** status** (Pos vs Neg)	0.321	0.011	0.899	0.878
**HPV risk groups** (only high risk-HPV, only low risk-HPV, both HPV types)	0.310	0.036	0.280	0.229

* Beta diversity analysis were performed with PERMANOVA and alpha diversity analysis with Kruskal-Wallis test.

** HPV types detected by the LiPA25 test: 6, 11, 16&, 18&, 31&, 33&, 34, 35&, 39&, 40, 42, 43, 44, 45&, 51&, 52&, 53, 54, 56&, 58&, 59&, 66, 68/73, 70, 74. (& high risk-HPV), (Labo Biomedical Products, Rijswijk, The Netherlands, based on licensed Innogenetics technology)
